# HLF and PPARα axis regulates metabolic‐associated fatty liver disease through extracellular vesicles derived from the intestinal microbiota

**DOI:** 10.1002/imt2.70022

**Published:** 2025-04-07

**Authors:** Xingzhen Yang, Jiale Wang, Xinyu Qi, Menglong Hou, Mengkuan Liu, Yang Xiao, Siqi Liu, Jinfeng Zhou, Jingsu Yu, Yang Wang, Guo Chen, Lin Yu, Khongorzul Batchuluun, Batbold Batsaikhan, Turtushikh Damba, Yuehui Liang, Xue Liang, Jie Ma, Yunxiao Liang, Yixing Li, Lei Zhou

**Affiliations:** ^1^ Guangxi Key Laboratory of Animal Breeding, Disease Control and Prevention, College of Animal Science and Technology Guangxi University Nanning China; ^2^ Institute of Digestive Disease Guangxi Academy of Medical Sciences, the People's Hospital of Guangxi Zhuang Autonomous Region Nanning China; ^3^ Wincon TheraCells Biotechnologies Co., Ltd. Nanning China; ^4^ Center for Research and Development of Institute of Biomedical Sciences Mongolian National University of Medical Sciences Ulaanbaatar Mongolia; ^5^ Department of Health Research, Graduate School Mongolian National University of Medical Sciences Ulaanbaatar Mongolia; ^6^ Department of Internal Medicine, Institute of Medical Sciences Mongolian National University of Medical Sciences Ulaanbaatar Mongolia; ^7^ School of Pharmacy Mongolian National University of Medical Sciences Ulaanbaatar Mongolia

**Keywords:** bile acids, extracellular vesicles, ferroptosis, gut microbiome, HLF, MAFLD

## Abstract

Metabolic‐associated fatty liver disease (MAFLD) has become increasingly widespread. The intestine is the primary site of lipid absorption and is important for the homeostasis of lipid metabolism. However, the mechanism underlying the participation of the intestinal tract in the development of MAFLD requires additional investigation. In this study, analysis of the single‐cell transcriptome of intestinal tissue from cynomolgus monkeys found that hepatic leukemia factor (HLF) participated in the genetic regulation of intestinal lipid absorption. Results obtained from normal and intestine‐specific *Hlf*‐knockout mice confirmed that HLF alleviated intestinal barrier disorders by inhibiting peroxisome proliferator‐activated receptor alpha (PPARα) expression. The HLF/PPARα axis alleviated MAFLD by mediating gut microbiota‐derived extracellular vesicles (fEVs), thereby inhibiting hepatocyte ferroptosis. Lipidomics and functional experiments verified that taurochenodeoxycholic acid (TCDCA), a conjugated bile acid contained in the fEVs, had a key role in the process. In conclusion, intestinal HLF activity was mediated by fEVs and identified as a novel therapeutic target for MAFLD.

## Highlights

- Intestinal‐specific lack of hepatic leukemia factor (HLF) can improve metabolic‐associated fatty liver disease (MAFLD).
- HLF plays a role in fat metabolism regulation through peroxisome proliferator‐activated receptor alpha (PPARα).
- The HLF/PPARα axis regulates the gut microbiome and influences the composition of gut microbiota‐derived extracellular vesicles (fEVs).
- The bound bile acid taurochenodeoxycholic acid (TCDCA) is a key regulatory factor for fEVs to improve MAFLD.

## Graphical abstract

The specific knockout of intestinal hepatic leukemia factor (HLF) improved metabolic‐associated fatty liver disease (MAFLD) by inhibiting peroxisome proliferator‐activated receptor alpha (PPARα) and restoring the intestinal barrier. Study of the mechanism revealed that the HLF/PPARα axis regulated gut microbiota‐derived extracellular vesicles (fEVs); which, through the gut‐liver cycle, suppressed hepatocyte ferroptosis and reduced hepatic steatosis. Lipidomics and functional assays identified the conjugated bile acid taurochenodeoxycholic acid (TCDCA) as the key driver of the lipid‐lowering effect of fEVs. The findings offer new therapeutic strategies for MAFLD.

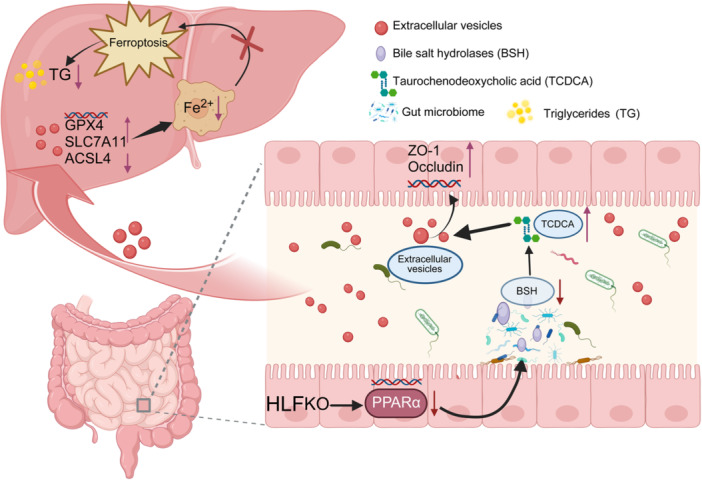

## INTRODUCTION

Metabolic‐associated fatty liver disease (MAFLD) is the most prevalent chronic liver disease worldwide, occurring in approximately 25% of the general population and in over 50% of individuals with metabolic dysfunction [1, 2, 3]. Despite extensive study of its pathogenesis, the molecular events underlying MAFLD are poorly understood.

The interplay between the liver and intestine in liver disease has emerged as a significant research interest [4]. Approximately 70% of the liver's blood supply is carried from the intestinal circulation by the portal vein. The portal circulation exposes the liver to the metabolites, endotoxins, and inflammatory mediators from intestinal microorganisms [5, 6]. The intestinal mucosal barrier serves as the primary physiological defense against exogenous pathogens, and the liver contributes to the maintenance of intestinal mucosal integrity by the secretion of specific antibodies and inflammatory factors [7, 8]. The physiological interaction between the liver and intestine is bidirectional. It involves the transport of bile and other active substances into intestinal lumen from the liver through bile ducts and transport of metabolites and other products from the intestine to the hepatic circulation by the portal vein following absorption by enterocytes [5]. The gut microbiota contributes to the maintenance of intestine‐liver interactions and immune homeostasis. Dysbiosis and compromised intestinal barrier function influence MAFLD progression [9, 10].

Recent studies have shown that extracellular vesicles (EVs) mediate intercellular communication, encapsulating molecules that have biological activity in adipose tissue, the liver, skeletal muscle, immune cells, and other targets [11, 12]. Bacterial‐derived EVs, or fecal EVs (fEVs) originate from the intestinal microbiota and interact with eukaryotic cells to modulate their functions. They encapsulate and transfer a variety of molecules, including proteins, enzymes, DNA, RNA, peptidoglycans, and lipids between and within tissues and function in ways similar to the EVs of eukaryotic cells [13, 14]. Study of the involvement of fEVs in metabolic processes is ongoing.

Hepatic leukemia factor (HLF) is a transcription factor [15]. Previous studies found that deletion of the *Tef*, *Dbp*, and *Hlf* genes in mice resulted in the development of cardiovascular disease, epilepsy, and accelerated aging, ultimately leading to a reduced lifespan [16, 17]. Furthermore, HLF expression in liver stellate cells is associated with the progression of liver fibrosis and promotes the onset and progression of liver cancer by activating c‐Jun [18, 19]. Previous studies found that olanzapine increased *Hlf* mRNA expression in vitro in a 3T3‐L1‐cell model of drug‐induced obesity [20]. Studies found a correlation between HLF and lipid metabolism. However, the mechanism by which HLF influences MAFLD is not clear.

Given the involvement of gut homeostasis in the pathogenesis of MAFLD and the importance of HLF for liver lipid metabolism, investigation in the context of MAFLD is crucial to adding to our understanding of the pathological progression of MAFLD. The findings will have significant implications for the development of novel MAFLD treatment strategies.

## RESULTS

### HLF is a novel target for regulating intestinal lipid absorption

**Figure 1:**
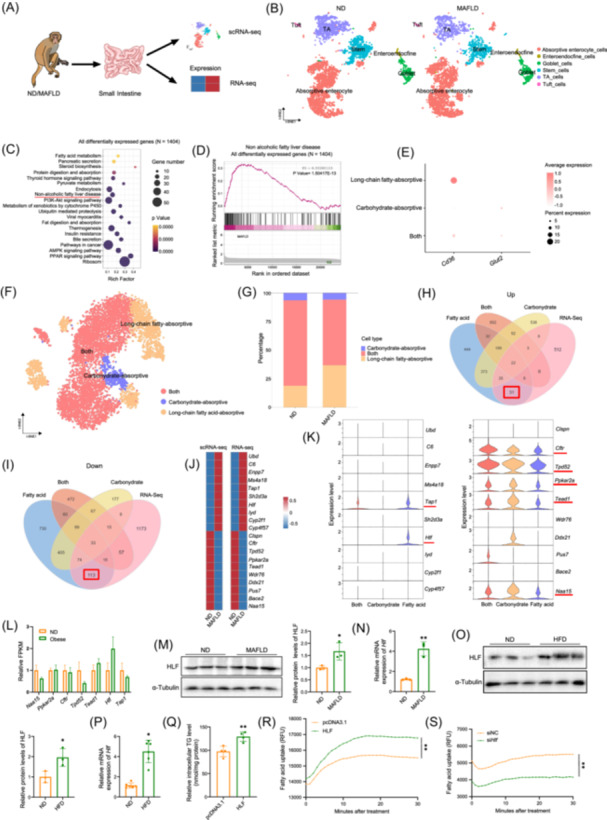
Hepatic leukemia factor (HLF) is a novel target for regulating intestinal lipid absorption. (A) Experimental workflow diagram. (B) Single‐cell sequencing annotation map of cell types in intestinal tissues from healthy and obese cynomolgus monkeys. (C, D) Kyoto encyclopedia of genes and genomes (KEGG) bubble plot and gene set enrichment analysis (GSEA) plot of all differentially expressed genes in intestinal absorptive epithelial cells (*N* = 1404). (E, F) Dimensionality reduction clustering bubble plot of marker genes and annotation map for intestinal absorptive epithelial cells. (G) Bar graph showing the proportion of cell types in both groups. (H, I) Venn diagrams of upregulated and downregulated genes in long‐chain fatty acid absorptive cells, carbohydrate absorptive cells, and dual‐function absorptive cells based on transcriptome data. (J) Heatmap of differentially expressed genes. (K) Violin plot of differentially expressed genes. (L) Bar graph showing the expression of differentially expressed genes in the intestinal transcriptome of cynomolgus monkeys. (M) Immunoblot and quantification of HLF protein in cynomolgus monkeys (*n* = 3). (N) Expression of *Hlf* mRNA in cynomolgus monkeys (*n* = 3). (O) Immunoblot and quantification of HLF protein in mice (*n* = 3). (P) Expression of *Hlf* mRNA in mice (*n* = 6). (Q) Intracellular triglyceride (TG) levels in Caco‐2 cells overexpressing HLF (*n* = 4). (R, S) Fatty acid uptake in HLF‐overexpressing and *Hlf*‐silenced cells (*n* = 4). Primer sequences are listed in Table S1. HFD, high‐fat diet group; MAFLD, metabolic‐associated fatty liver disease; ND, control group. Data are presented as mean ± standard deviation. Repeated measures analysis of variance was used to compare trends across two curves over multiple time points. *p < 0.05, **p < 0.01.

Both healthy cynomolgus monkeys and those with spontaneous MAFLD who were 25–28 years of age, equivalent to 70–80 years in humans, were selected after long‐term monitoring of their health status (Figure S1A). Analysis of single‐cell RNA sequencing (scRNA‐seq) and transcriptome sequencing (RNA‐seq) of intestinal tissue from both groups of monkeys revealed the distribution of six epithelial cell types (Figure 1A,B and Figure S1B). The percentages of absorptive cells, endocrine cells, and goblet cells were increased in the MAFLD group, and the percentages of stem cells, transit‐amplifying cells, and tuft cells were decreased (Figure S1C). Intestinal epithelial cells assume a pivotal role in the absorption of nutrients such as fatty acids, amino acids, and glucose. Consequently, we elected to undertake an analysis of the absorptive intestinal epithelial cells (Figure S1D). Kyoto Encyclopedia of Genes and Genomes (KEGG) functional enrichment analysis of the differentially expressed genes (DEGs) disclosed significant correlations with pathways related to nonalcoholic fatty liver disease, fatty‐acid metabolism, and peroxisome proliferator‐activated receptor (PPAR) signaling (Figure 1C,D). The absorptive cells included types that long‐chain fatty acids, carbohydrates, and cells that absorb both (Figure 1E,F). The percentage of long‐chain fatty‐acid‐absorbing cells increased, and the percentage of carbohydrate‐absorbing cells decreased in the cynomolgus monkeys with MAFLD (Figure 1G). A Venn diagram was constructed for the DEGs of the three absorptive cell types and the DEGs of the transcriptome (Figure 1H,I). Heatmaps of the top 10 upregulated and downregulated genes in absorptive cells (Figure 1J,K) show that only *Hlf* was highly expressed in epithelial cells associated with the absorption of long‐chain fatty acids. Furthermore, the transcriptome analysis of cells from the intestines of obese cynomolgus monkeys that was available from a public database also indicated that *Hlf* was upregulated [21], which is consistent with our results (Figure 1L). The data indicate that HLF has a key role in the absorption of intestinal lipids. The changes in HLF expression in the intestinal cells of both groups of cynomolgus monkeys were confirmed by protein immunoblotting and quantitative real‐time reverse polymerase chain reaction (qPCR) (Figure 1M,N and Tables S1–S2) and were consistent with the sequencing results. We subsequently established a mouse model of MAFLD and discovered that expression of *Hlf* mRNA and protein in the intestine were significantly upregulated by feeding a high‐fat diet (HFD) (Figure 1O,P and Tables S1–S2). Furthermore, overexpression of HLF in Caco‐2 cells resulted in a significant increase in triglycerides (TG), an increase in lipid droplets shown by Oil‐red O staining, and increased fatty‐acid uptake. The expected results were obtained upon silencing *Hlf* (Figure 1Q–S and Figure S1E–K). These findings show that HLF influenced intestinal lipid absorption.

### HLF deficiency improves MAFLD

**Figure 2:**
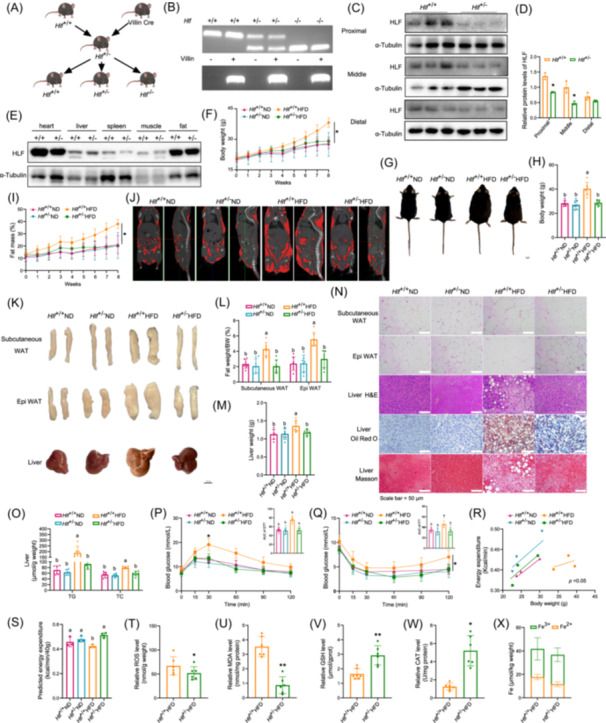
Partial hepatic leukemia factor (HLF) deficiency improves metabolic‐associated fatty liver disease (MAFLD). (A) Schematic diagram of breeding for intestinal‐specific heterozygous *Hlf* knockout mice. (B) Genotype Identification of mice. (C, D) Western blot analysis of HLF expression in intestinal tissues (*n* = 3). (E) Western blot analysis of HLF expression in the heart, liver, spleen, muscle, and adipose tissues (*n* = 3). (F–H) Body weight and representative photographs of mouse body size at the end of the experiment (*n* = 6). (I) Body fat percentage of mice (*n* = 6). (J) Computed tomography (CT) imaging of mice. (K–M) Epididymal fat, subcutaneous fat, and liver weights along with corresponding tissue weights (*n* = 7). (N) H&E staining of epididymal and subcutaneous fat, and H&E, Oil Red O, and Masson staining of the liver. (O) Liver triglyceride (TG) and total cholesterol (TC) levels (*n* = 6). (P) Glucose tolerance test (GTT) and quantification of AUC in mice (*n* = 6). (Q) Insulin sensitivity test and quantification of AUC in mice (*n* = 6). (R, S) Energy metabolism analysis normalized to 40 g body weight (*n* = 3). (T–X) Liver levels of reactive oxygen species (ROS), malondialdehyde (MDA), glutathione (GSH), catalase (CAT), and Fe²⁺/Fe³⁺ (*n* = 6–8). Data are presented as mean ± standard deviation. The Friedman test was used for four‐group comparisons with repeated measures over time. *p < 0.05, **p < 0.01. Different letters in the figure indicate significant differences between groups.

To explore the role of intestinal HLF in disorders of liver lipid metabolism, we bred *Hlf* knockout mice with heterozygous intestine‐specific expression (*Hlf* ^+/−^; Figure 2A). Genotype characterization confirmed successful breeding (Figure 2B). HLF expression was decreased in all sections of the small intestine of *Hlf* ^+/−^ mice (Figure 2C,D and Table S2) and there was no change in expression in the liver, heart, and other organs (Figure 2E). The wild‐type and intestine‐specific *Hlf* ^+/−^ mice were given a normal diet or an HFD for 12 weeks. In the HFD group, body weight and body‐fat percentage were significantly reduced in the *Hlf* ^+/−^ mice (Figure 2F–I) and the percentage of lean meat was significantly increased (Figure S2A). There was no difference in food intake (Figure S2B). Microcomputed tomography indicated that fat accumulation in *Hlf* ^+/−^ mice was significantly decreased (Figure 2J). We also found that liver lipid accumulation and liver volume and weight were decreased in *Hlf* ^+/−^ mice, and the liver had a redder appearance (Figure 2K–M). Histological staining confirmed that *Hlf* ^+/−^ mice had reduced liver lipid accumulation and decreased fat cell size (Figure 2N). Compared with HFD‐fed control mice, TG and TC levels in the liver of *Hlf* ^+/−^ mice were lower (Figure 2O), and the relevant serum markers were improved (Figure S2C–H), indicating that *Hlf* deficiency protected against the lipid accumulation and liver injury induced by the HFD. To evaluate the influence of *Hlf* on glucose metabolism, we performed glucose tolerance tests (GTT) and insulin tolerance tests (ITT). The results revealed that the *Hlf* ^+/−^ mice had decreased fasting blood glucose, enhanced glucose tolerance, and increased insulin sensitivity (Figure 2P,Q and Figure S2I). Metabolic cage tests found that O_2_ consumption and CO_2_ production were significantly improved in the *Hlf* ^+/−^ mice fed an HFD, no significant change was observed in respiratory exchange ratio, and energy consumption increased (Figure 2R,S and Figure S2J–P). These findings imply that *Hlf* knockout alleviated the metabolic changes induced by HFD by increasing energy metabolism. Investigation of the impact of HLF on oxidative stress found that production of reactive oxygen species (ROS), malondialdehyde (MDA), and lipid peroxides/lactoperoxidase (LPO) activity in the liver and induced by the HFD were decreased in the *Hlf* ^+/−^ mice (Figures 2T,U, and S2Q), glutathione (GSH), catalase (CAT), superoxide dismutase (SOD), mitochondrial membrane potential (JC‐1) and adenosine triphosphate (ATP) level were increased (Figures 2V,W and S2R–T), and Fe^2+^ content was decreased (Figure 2X). Meanwhile, the protein level of the ferroptosis marker glutathione peroxidase 4 (GPX4) was increased (Figure S2U). The results indicate that *Hlf* ^+/−^ reduced oxidative stress and inhibited ferroptosis. Meanwhile, results demonstrating that intestine‐specific *Hlf* knockout improved MAFLD were obtained in homozygous *Hlf*‐knockout mice (Figures S3, S4).

### Intestinal *Hlf* deficiency improves lipid digestion and absorption in mice

The influence of HLF on lipid digestion and absorption was investigated by oral administration of olive oil. After treatment, serum free fatty‐acid (FFA) and TG levels were significantly lower in *Hlf* ^+/−^ mice than in *Hlf* ^+/+^ mice (Figure S5A–D). Intraperitoneal administration of the lipase inhibitor tyloxapol followed by olive oil gavage in mice fed the HFD resulted in significantly lower serum FFA and TG in *Hlf* ^+/−^ mice than in *Hlf* ^+/+^ mice (Figure S5E–H), indicating that the deletion of HLF inhibited the digestion and absorption of exogenous lipids. The serum lipopolysaccharide (LPS), cluster of differentiation 14 (sCD14), and lipopolysaccharide binding protein (LBP) concentrations were also lower in *Hlf* ^+/−^ mice than in *Hlf* ^+/+^ mice (Figure S5I,J). Hematoxylin and eosin and Oil‐red O staining of various regions of the small intestine revealed a marked reduction of villus area and decrease of lipid content in the *Hlf* ^+/−^ mice (Figure S5K,L). *Hlf* deficiency also resulted in increased expression of the intestinal tight junction proteins zonula occludens‐1 (ZO‐1) and occludin (Figure S5M–Q), indicating that *Hlf* deficiency may also inhibit the digestion and absorption of exogenous lipids by enhancing the intestinal barrier.

### HLF regulates the expression of *Ppara*

**Figure 3:**
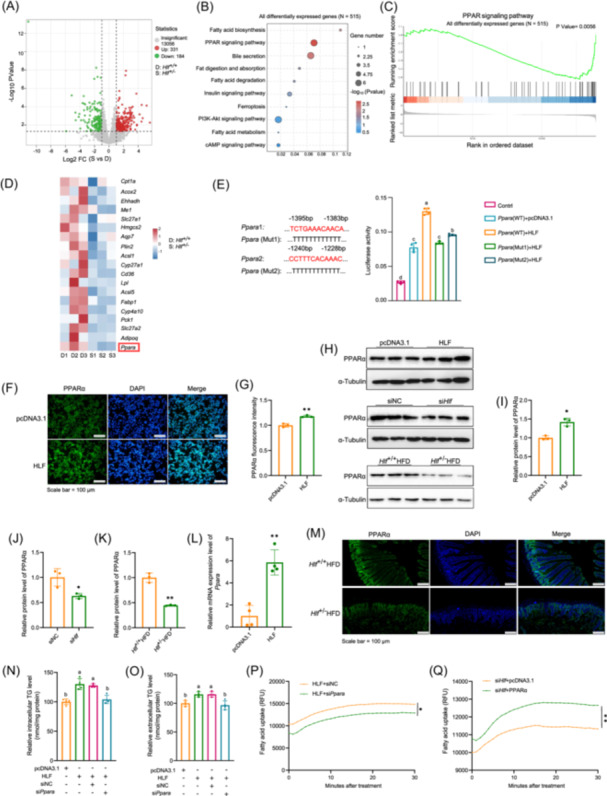
Hepatic leukemia factor (HLF) regulates peroxisome proliferator‐activated receptor alpha (PPARα) expression. (A) Volcano plot of differentially expressed genes (S vs. D). D correspond to *Hlf* ^+/+^, S correspond to *Hlf* ^+/−^. (B) Kyoto encyclopedia of genes and genomes (KEGG) enrichment analysis of all differentially expressed genes (*N* = 515). (C) Gene set enrichment analysis (GSEA) of all differentially expressed genes (*N* = 515). (D) Heatmap of differentially expressed genes. D1–D3 correspond to *Hlf* ^+/+^1–*Hlf* ^+/+^3, and S1–S3 correspond to *Hlf* ^+/^^−^1–*Hlf* ^+/^^−^3. (E) *Ppara* promoter activity assay (*n* = 4). (F, G) Immunofluorescence analysis and quantification of PPARα expression in Caco‐2 (*n* = 3). (H–K) Western blot analysis and quantification of PPARα expression in Caco‐2 and intestinal tissues of mice (*n* = 3). (L) *Ppara* mRNA levels in Caco‐2 overexpressing HLF (*n* = 4). (M) Immunofluorescence analysis of PPARα in mouse intestinal tissues. (N, O) Intracellular and extracellular triglyceride (TG) levels (*n* = 4). (P, Q) Fatty acid uptake levels (*n* = 4). Data are presented as mean ± standard deviation. Repeated measures analysis of variance was used to compare trends across two curves over multiple time points. *p < 0.05, **p < 0.01. Different letters in the figure indicate significant differences between groups.

To further clarify the mechanism of action of HLF, we performed RNA sequencing of jejunal tissues from *Hlf* ^+/+^ and *Hlf* ^+/−^ mice fed an HFD (Figure 3A). KEGG analysis of DEGs revealed significant enrichment of the PPAR signaling pathway, fatty‐acid synthesis and metabolism, and fat digestion and absorption. Gene set enrichment analysis (GSEA) showed that HLF expression was positively correlated with PPAR signaling (Figure 3B,C). The gene expression analysis showed that *Hlf* deficiency led to the downregulation of *Ppara* and its target genes *Fabp1*, *Cyp4a10*, and *Cpt1a*, indicating that HLF regulated *Ppara* expression (Figure 3D). Subsequently, the predicted HLF binding sites within the *Ppara* promoter were mutated, and the transcriptional activity of the *Ppara* promoter was detected by a dual luciferase reporter system. This result indicated that these mutations suppressed the transcriptional activity of the *Ppara* promoter, thereby showing that HLF was involved in the transcriptional regulation of *Ppara* (Figure 3E). Immunofluorescence, western blot analysis, and qPCR demonstrated that HLF promoted the expression of *Ppara* and that silencing *Hlf* inhibited its expression (Figure 3F–M and Tables S1–S2). Silencing *Ppara* reduced the uptake of TG and fatty acids mediated by HLF and PPARα overexpression blocked the downregulatory effect of silenced *Hlf* on TG and fatty acid uptake (Figure 3N–Q). These outcomes are consistent with HLF regulation of intestinal lipid metabolism via PPARα.

### Inhibition of PPARα alleviates MAFLD

**Figure 4:**
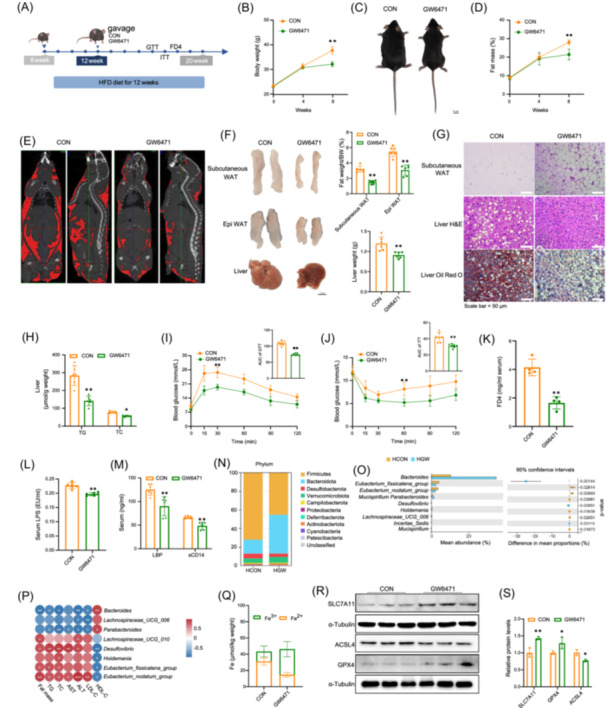
Inhibition of peroxisome proliferator‐activated receptor alpha (PPARα) alleviates metabolic‐associated fatty liver disease (MAFLD). (A) Schematic diagram of the mouse experimental design. (B, C) Mouse body weight and representative images of body size (*n* = 6). (D) Body fat percentage of mice (*n* = 6–7). (E) Computed tomography (CT) imaging of mice. (F) Epididymal fat, subcutaneous fat, and liver weights, along with representative images (*n* = 6). (G) H&E staining of subcutaneous fat, and H&E and Oil Red O staining of liver tissue. (H) Liver triglyceride (TG) and total cholesterol (TC) levels (*n* = 6). (I) Glucose tolerance test (GTT) and quantification of AUC (*n* = 6). (J) Insulin tolerance test (ITT) and quantification of AUC (*n* = 6). (K) Serum FITC‐dextran 4 (FD4) levels in mice (*n* = 4). (L–M) Serum levels of lipopolysaccharide (LPS), lipopolysaccharide‐binding protein *(*LBP), and cluster of differentiation 14 (sCD14) in mice (*n* = 6). (N) Proportional analysis of gut microbiota at the phylum level. (O) Differential species between the HCON and HGW groups. (P) Correlation analysis of gut microbial species with mouse phenotypic traits. (Q) Liver Fe²⁺/Fe³⁺ levels (*n* = 6–8). (R, S) Western blot analysis and quantification of hepatic solute carrier family 7 member 11 (SLC7A11), glutathione peroxidase 4 (GPX4), and acyl‐coa synthetase long chain family member 4 (ACSL4) expression (*n* = 3). Data are presented as mean ± standard deviation. Repeated measures analysis of variance was used to compare trends across two curves over multiple time points. *p < 0.05, **p < 0.01.

A PPARα inhibitor (GW6471) was used to investigate whether PPARα participated in the regulation of HLF in a mouse MAFLD model. Viability assays found that 10 μmol GW6471 was not toxic in Caco‐2 cells (Figure S6A) and it decreased intracellular TG level (Figure S6B). Subsequently, after feeding 8‐week‐old mice an HFD for 4 weeks, a control group was given normal saline, and an experimental group was given GW6471 for 8 weeks (Figure S7A,B and Figure 4A). At 8 weeks, the body weight and body‐fat percentage of the mice in the GW6471 group were significantly reduced compared with controls (Figures 4B–D and S6C), the lean ratio (Figure S6D) was significantly increased, and there was no difference in food intake (Figure S6E). Micro‐CT found that fat accumulation in the organs of mice treated with GW6471 was decreased (Figure 4E). Hepatic lipid accumulation, liver volume, and weight were lower than in the controls, and the color of the liver was redder (Figure 4F). Staining of liver tissue confirmed that GW6471 decreased hepatic lipid accumulation and adipocyte size (Figure 4G). Compared with control mice, TG and total cholesterol (TC) levels were lower in the livers of mice treated with GW6471 (Figure 4H). All related serum indicators were improved (Figure S6F–I), suggesting that inhibition of PPARα decreased the lipid deposition and liver injury induced by the HFD. The GTT and ITT results revealed that GW6471 decreased fasting blood glucose and increased glucose tolerance, and insulin sensitivity (Figures 4I,J and S6J). The metabolic cage results showed that O_2_ consumption and CO_2_ production of mice in the GW6471 group were significantly elevated, indicating increased energy expenditure (Figure S6K–R). The influence of PPARα on the intestinal barrier was estimated by oral administration of 4 kDa fluorescein isothiocyanate‐dextran. Serum FD4, LPS, LBP, and sCD14 were all reduced in mice in the GW6471 group (Figure 4K–M) and expression of the tight junction proteins ZO‐1 and occludin in the intestinal was significantly increased (Figure S6S). The results show that inhibition of PPARα improved the intestinal barrier function of mice fed an HFD. Feces were collected for 16S rDNA sequencing to assess variations in the intestinal microbiota. The principal coordinates analysis plot (Figure S6T) shows the distribution of samples from the two groups. The analysis shows that phylum Firmicutes declined and phylum Bacteroidetes increased in the GW6471 (HGW) group (Figure 4N). Previous studies have reported that an increased Firmicutes/Bacteroidetes ratio was associated with obesity [22], which is consistent with our results. At the genus level, enrichment analysis found increases in the abundance of beneficial genera such as *Bacteroides*, *Parabacteroides*, and *Lachnospiraceae*_UCG‐006 in the HGW group. The abundance of harmful genera such as the *Eubacterium_fissicatena_group*, *Desulfovibrio*, and *Holdemania* were reduced (Figure 4O). The correlations of various bacterial genera and the in vivo serum phenotype were significant (Figure 4P). Prediction of the functions of the intestinal microbiota indicated that inhibition of PPARα improved sugar metabolism, vitamin metabolism, lipid metabolism, and steroid and ketone metabolism (Figure S6U,V). This implies that inhibition of PPARα ameliorated the extent of change of intestinal microbiota. We also found that inhibition of PPARα by GW6471 reduced oxidative stress by decreasing ROS production, MDA, and LPO activity (Figures S6W,X and S7C), increasing GSH, CAT, SOD, ATP, and JC‐1 level (Figures S6Y,Z and S7D–F), and decreasing Fe^2+^ (Figure 4Q) in the liver. The expression of the ferroptosis‐related proteins solute carrier family 7 member 11 (SLC7A11) and GPX4 was increased and that of acyl‐coa synthetase long chain family member 4 (ACSL4) was decreased (Figure 4R,S and Table S2). The results show that HLF alleviated MAFLD by inhibiting PPARα signaling, which decreased oxidative stress and improved the homeostasis of the intestinal microbiota.

### The HLF/PPARα axis regulates lipid metabolism through fEVs

As EVs play a crucial role in enterohepatic circulation, we treated mice with GW6471 and isolated fEVs from the intestinal contents to compare the effects on MAFLD in treated and control mice. Transmission electron microscopy and nanoparticle tracking analysis found that the size of the fEVs from GW6471‐treated and control mice were comparable, but there were more fEVs in the samples from treated mice (Figure S8A). Detection of the eukaryote‐specific EV markers, TSG101, CD9, CD81, and the intestine‐specific marker GPA33 verified the successful isolation of fEVs (Figure S8B). Endocytosis assays showed that HepG2 cells internalized fEVs (Figure S8C) and fEVs from GW6471‐treated mice reduced intracellular and extracellular TG in both primary hepatocytes and HepG2 cells (Figures S8D,E and S9A,B). The fEVs from GW6471‐treated mice reduced fatty‐acid uptake and the uptake of 4 kDa and 40 kDa fluorescein isothiocyanate‐dextran, and increased the expression of occludin and ZO‐1 proteins (Figure S8F–I), all of which indicate changes in intestinal permeability. Transwell coculture of HepG2 and Caco‐2 cells indicated that fEVs from GW6471‐treated mice increased the viability hepatocytes (Figure S9C), which indicates that the regulatory activity of fEVs involved translocation to the liver. Further study revealed that these fEVs increased the number of mitochondria, and GSH, SOD, and CAT levels in HepG2 and primary liver cells (Figures S8J,K and S9D,E) and reduced MDA and ROS levels, and production of mitochondrial ROS (Figures S8L,M and S9F,G). The fEVs from treated mice decreased Fe^2+^ content and lipid peroxidation and increased calcein fluorescence and the mitochondrial membrane potential (Figures S8N–R and S9H–J). Western blot analysis demonstrated that they changed the expression of ferroptosis‐associated proteins (Figure S8S). Meanwhile, fEVs were extracted from the mice with intestinal‐specific knockout of *Hlf*, and biochemical experiments were carried out in primary cells, which were consistent with the expected results (Figure S9K–P). The study results suggest that both fEVs from GW6471‐treated and *Hlf* ^+/−^ fEVs decreased liver steatosis by improving intestinal barrier function and suppressing ferroptosis.

### fEVs from GW6471‐treated mice improve intestinal permeability and inhibit ferroptosis

**Figure 5:**
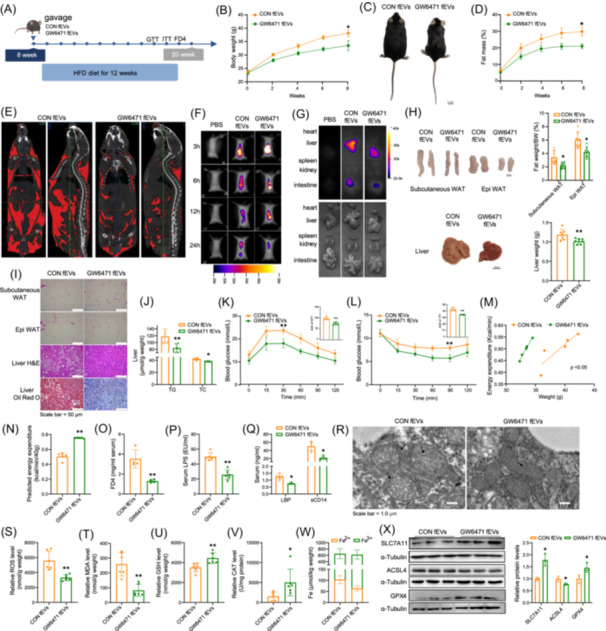
Gut Microbiota‐derived extracellular vesicles (fEVs) from GW6471‐treated mice improve intestinal permeability and inhibit ferroptosis. (A) Schematic diagram of the mouse experimental procedure. (B, C) Body weight curve and representative photos of mouse body morphology (*n* = 6). (D) Body fat percentage of mice (*n* = 6). (E) Computed tomography (CT) imaging of mice. (F, G) In vivo and tissue tracing of fluorescence‐labeled fEVs in mice (*n* = 3). (H) Weight of epididymal fat, subcutaneous fat, and liver in mice (*n* = 6). (I) H&E staining of adipose tissue, H&E staining, and Oil Red O staining of liver tissue. (J) Liver triglyceride (TG) and total cholesterol (TC) levels (*n* = 6). (K) Glucose tolerance test and area under the curve (AUC) quantification in mice (*n* = 6). (L) Insulin tolerance test and AUC quantification in mice (*n* = 6). (M, N) Energy metabolism analysis and energy expenditure normalized to 40 g in mice (*n* = 5). (O) Serum FD4 levels in mice (*n* = 4). (P, Q) Serum lipopolysaccharide (LPS), lipopolysaccharide‐binding protein (LBP), and cluster of differentiation 14 (sCD14) levels in mice (*n* = 6). (R) Transmission electron microscopy (TEM) images of the liver. The arrows indicate mitochondrial cristae. (S–W) Levels of reactive oxygen species (ROS), Malondialdehyde (MDA), glutathione (GSH), catalase (CAT), and Fe²⁺/Fe³⁺ in the liver (*n* = 6). (X) Western blot analysis and quantification of solute carrier family 7 member 11 (SLC7A11) and acyl‐coa synthetase long chain family member 4 (ACSL4) in mouse liver (*n* = 3). Data are presented as mean ± standard deviation. Repeated measures analysis of variance was used to compare trends across two curves over multiple time points. *p < 0.05, **p < 0.01.

To evaluate the in vivo effects of fEVs, 8‐week‐old wild‐type mice were given control and GW6471 fEVs by gavage and fed them an HFD for 12 weeks (Figure 5A). The body weight and body‐fat percentage of mice given the GW6471 fEVs were significantly decreased, and the percentage of lean meat was significantly elevated compared with the control mice, but their food consumption was not different (Figures 5B–D and S10A–C). Micro‐CT evaluation clearly revealed that the GW6471 fEVs reduced fat accumulation (Figure 5E). In vivo tracking showed that the fEVs reached the liver in approximately 6 h and that the signal was decreased after 24 h (Figure 5F). Tissue imaging showed that the fEVs accumulated in the liver and intestine (Figure 5G). Treatment with GW6471 fEVs decreased the volume and weight of fat and of the liver and sustained the ruddy color of the liver (Figure 5H). Staining of tissue sections showed that GW6471 fEVs decreased liver steatosis and adipocyte size (Figure 5I). Furthermore, relevant liver and serum indicators improved (Figures 5J and S10D–H), indicating that GW6471 fEVs improved lipid metabolism abnormalities and reduced liver injury induced by the HFD. The GTT and ITT results showed that GW6471 fEVs decreased fasting blood glucose, and increased glucose tolerance and insulin sensitivity (Figures 5K,L and S10I). The metabolic cage results indicated that GW6471 fEVs significantly increased energy expenditure, with increases of O_2_ consumption and CO_2_ production (Figures 5M,N and S10J–O). The GW6471 fEVs also significantly decreased serum FD4, LPS, LBP, and sCD14 levels (Figure 5O–Q) and increased the expression of occludin, a tight junction protein in intestinal tissue (Figure S10P), consistent with improvement of the intestinal barrier. Evaluation of mitochondrial morphology by transmission electron microscopy found the mitochondrial membranes were more intact, and the mitochondrial cristae were partially restored by the GW6471 fEVs (Figure 5R). GW6471 fEVs reduced ROS, MDA, and LPO levels (Figures 5S,T and S10Q), increased of GSH, CAT, and SOD content (Figures 5U,V and S10R), and decreased the Fe^2+^ content (Figure 5W) of the liver. Western blot analysis revealed that the expression of the ferroptosis‐related protein SLC7A11 and GPX4 was increased in the liver, and that of ACSL4 was decreased (Figure 5X). The findings demonstrate that fEVs from GW6471‐treated mice alleviated MAFLD by improving the intestinal barrier and inhibiting ferroptosis.

### Lipids in fEVs affect hepatic steatosis

**Figure 6:**
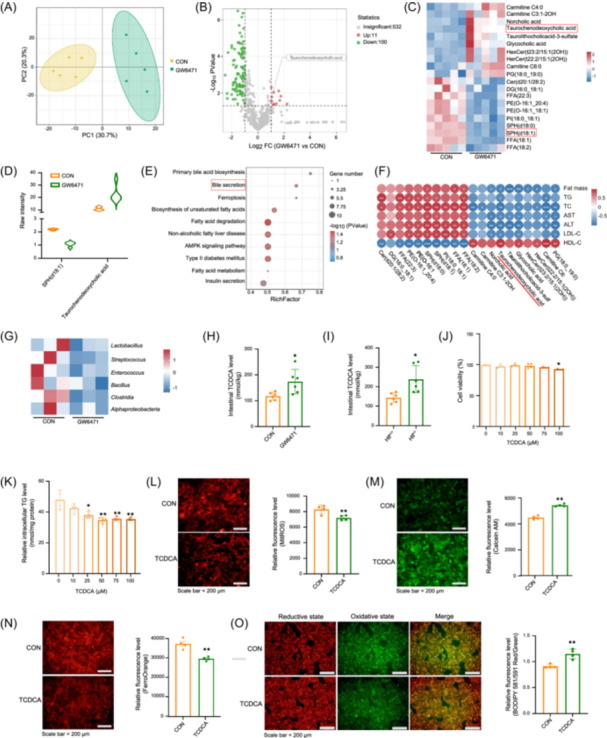
Lipid Alterations in gut microbiota‐derived extracellular vesicles (fEVs) influence hepatic steatosis. (A) PCA plot of lipidomics data. (B) Volcano plot of differential lipid species in the lipidomics analysis (GW6471 vs CON). (C) Heatmap of differentially abundant lipids. (D) Violin plots of SPH (d18:1) and taurochenodeoxycholic acid (TCDCA) levels. (E) Kyoto Encyclopedia of Genes and Genomes (KEGG) pathway enrichment analysis. (F) Heatmap showing the correlation between lipidomics data and in vivo phenotypes. (G) Heatmap of bacteria enriched with BSH enzymes. (H) TCDCA levels in intestinal contents of CON and GW6471‐treated mice (*n* = 6). (I) TCDCA levels in intestinal contents of *Hlf* ^+/+^ and *Hlf* ^+/^^−^ mice (*n* = 6). (J) Cell viability assay (CCK8) in HepG2 cells. (K) Intracellular TG levels in HepG2 cells treated with TCDCA (*n* = 4). (L–O) Fluorescence images and quantification of mitochondrial ROS (MitROS), Calcein‐AM (calcium green), FerroOrange, and lipid peroxidation in HepG2 cells (*n* = 4). Data are presented as mean ± standard deviation. *p < 0.05, **p < 0.01.

The effects of fEVs on lipid metabolism in the liver were investigated by lipidomic analysis of control and GW6471 fEVs. The results indicated that, compared with the controls, 11 lipids were upregulated, and 100 were downregulated in the GW6471 fEVs (Figure 6A,B). A clustered heatmap of the top 20 differentially expressed lipids (Figure 6C,D) shows that the cirrhosis marker SPH (d18:1) was significantly downregulated, and secondary bile acids (BAs) such as taurochenodeoxycholic acid were significantly upregulated. KEGG enrichment analysis indicated strong enrichment in pathways such as bile acid secretion, nonalcoholic fatty liver, fatty‐acid degradation, and type 2 diabetes (Figure 6E). Analysis of the correlation of differentially expressed lipids and mouse phenotypes (Figure 6F), revealed that TCDCA was significantly associated with phenotypes and serum indicators. Subsequent heatmap analysis (Figure 6G) showed that bacteria associated with intestinal bile acid decomposition were downregulated. These bacteria contain bile salt hydrolase (BSH), which converts BAs conjugated to taurine into nonconjugated BAs. A decrease of BSH‐positive bacteria may increase TCDCA in the intestine. In this study, the amount of TCDCA in the intestinal contents in mice increased when PPARα activity was inhibited or *Hlf* was deficient (Figure 6H,I). This is consistent with the lipidomics results and is responsible for the increase in TCDCA in fEVs. Treatment of HepG2 cells with varying concentrations of TCDCA demonstrated no cytotoxicity in the CCK‐8 assay, with the most significant reduction in TG levels observed at 50 μM. (Figure 6J,K). Functional experiments showed that TCDCA reduced oxidative stress and inhibited ferroptosis (Figure 6L–O), implying that it is involved in fEVs mediated regulation of liver lipid metabolism.

## DISCUSSION

In this study, we found that intestine‐specific knockout of *Hlf* ameliorated MAFLD. The knockout of *Hlf* led to the downregulation of its downstream target gene *Ppara*, which improved the gut microbiota composition. EVs derived from the gut microbiota, mitigated intestinal barrier damage and inhibited ferroptosis through the gut‐liver axis, thereby alleviating hepatic steatosis. Furthermore, we also identified TCDCA as a key regulatory factor within EVs (Movie S1).

HLF, is a member of the bZIP transcription factor family (PAR bZIP) [23], and is closely linked to cancer progression [24]. Studies have shown that HLF overexpression enhances sorafenib resistance in hepatocellular carcinoma [25] and promotes the proliferation and metastasis of triple‐negative breast cancer (TNBC) cells [26]. In addition, HLF also participates in lipid metabolism, with significant downregulation of *Hlf* mRNA in the livers of mice with reduced lipid accumulation and weight loss [27]. Knockdown of HLF in 3T3‐L1 cells also reduces lipid levels [20]. However, the mechanisms underlying HLF‐mediated lipid regulation remain unclear. In this study, RNA sequencing revealed that *Hlf* deficiency in intestinal tissue significantly enriched DEGs in the PPAR signaling pathway (Figure 3D). Dual luciferase assays confirmed that HLF transcriptionally regulated PPARα (Figure 3E). Functionally, pharmacological inhibition of PPARα blocked the effects of HLF, but PPARα overexpression reversed the suppression induced by *Hlf* deficiency (Figure 3N–Q). These findings identify *Ppara* as a downstream target of HLF, highlighting its role in regulating HLF‐mediated lipid metabolism.

The gut microbiota is recognized as a key regulator of host metabolism. In recent years, fEVs have gained increasing research attention [28]. Studies show that fEVs help maintain intestinal barrier integrity and influence metabolic health. In MAFLD and MASH patients, fEVs are linked to barrier dysfunction, inflammation, and liver injury [29]. Additionally, fEVs from HFD‐fed mice induce insulin resistance in skeletal muscle and adipocytes [30], and *Akkermansia muciniphila*‐derived EVs improve gut permeability and metabolic function in diabetic mice [31]. Here, we found that PPARα inhibition in the intestine increased the abundance of beneficial bacteria and reduced that of harmful bacteria (Figure 4N). Both in vitro and in vivo, fEVs from HLF or PPARα suppression improved gut permeability and alleviated hepatic steatosis. These findings underscore fEVs as key modulators of gut microbiota‐host interactions in hepatic lipid metabolism.

Bile acids (BAs) are key regulators of the gut‐liver axis [32], with distinct physiological functions. For example, cholic acid (CA) increases the Firmicutes/Bacteroidetes ratio in normal‐diet mice, leading to metabolic dysregulation [33], whereas hyodeoxycholic acid (HDCA) alleviates MAFLD via the gut‐liver axis [34]. TCDCA improves hyperlipidemia by modulating glycerophospholipid metabolism [35]. Gut microbiota utilizes BSH to deconjugate bile acids, influencing primary bile acid metabolism. Studies show that HFD‐fed mice supplemented with Pu‐erh tea exhibit reduced BSH activity, leading to conjugated bile acid accumulation and improved obesity and steatohepatitis [36]. In this study, PPARα inhibition significantly reduced BSH‐rich gut microbiota (Figure 6G). Lipidomic analysis revealed increased TCDCA levels in fEVs from *Hlf*‐deficiency and PPARα‐inhibited groups (Figure 6H,I). These findings suggest that HLF and PPARα suppression promote TCDCA accumulation by reducing gut BSH activity, underscoring the role of gut microbiota in primary bile acid metabolism and lipid homeostasis.

Iron metabolism is essential for maintaining physiological homeostasis, and iron overload is considered a key factor in MAFLD progression [37]. Excess intracellular ferrous ions induce oxidative stress, leading to metabolic dysregulation and exacerbation of MAFLD development [38]. Studies have shown that EVs regulate ferroptosis and influence disease progression. For example, EVs derived from mesenchymal stem cells alleviate acute liver injury by inhibiting hepatocyte ferroptosis [39], while EVs from Clostridium difficile modulate metabolic activity by increasing intracellular ROS and reducing mitochondrial membrane potential [40]. In this study, fEVs from *Hlf*‐deficiency or PPARα‐inhibited models effectively mitigated oxidative stress and suppressed ferroptosis. Correlation analysis between differential lipids and gut microbiota identified TCDCA as the key bioactive component within fEVs (Figure 6F). Based on these findings, we propose that gut microbiota‐derived EVs influence ferroptosis through their lipid components.

## CONCLUSION

In summary, our study shows that the HLF/PPARα axis regulated the gut‐liver cycle and hepatic ferroptosis via gut microbiota‐derived EVs. The conjugated bile acid TCDCA was identified as the key mediator of the lipid‐lowering activity of fEVs. These findings clarify the function of intestinal HLF in regulating MAFLD and offer new therapeutic perspectives for treating the disease.

## METHODS

The details of the materials and methods used in this study and statistical analysis are included in online supplemental materials.

## AUTHOR CONTRIBUTIONS

**Xingzhen Yang, Yixing Li,** and **Lei Zhou:** Conceptualization. **Xingzhen Yang, Jiale Wang, Xinyu Qi, Menglong Hou,** and **Mengkuan Liu:** Formal analysis. **Yixing Li** and **Lei Zhou:** Funding acquisition. **Xingzhen Yang:** Data curation. **Yixing Li** and **Lei Zhou:** Project administration. **Xingzhen Yang, Jiale Wang, Xinyu Qi, Yang Xiao, Siqi Liu, Yixing Li,** and **Lei Zhou:** Methodology. **Xingzhen Yang, Jiale Wang,** and **Menglong Hou:** Validation. **Xingzhen Yang, Jiale Wang,** and **Xinyu Qi:** Software. **Xingzhen Yang, Jiale Wang, Yixing Li,** and **Lei Zhou:** Visualization. **Xingzhen Yang, Jiale Wang, Xinyu Qi, Menglong Hou, Mengkuan Liu, Yang Xiao, Siqi Liu, Jinfeng Zhou, Yang Wang, Jingsu Yu, Guo Chen, Lin Yu, Khongorzul Batchuluun, Batbold Batsaikhan, Turtushikh Damba, Yuehui Liang,** and **Xue Liang:** Investigation. **Turtushikh Damba, Yuehui Liang, Xue Liang, Jie Ma, Yunxiao Liang, Yixing Li,** and **Lei Zhou:** Supervision. **Xingzhen Yang** and **Lei Zhou:** Writing–original draft. **Xingzhen Yang, Jiale Wang, Xinyu Qi, Menglong Hou, Mengkuan Liu, Yang Xiao, Siqi Liu, Jinfeng Zhou, Jingsu Yu, Yang Wang, Guo Chen, Lin Yu, Khongorzul Batchuluun, Batbold Batsaikhan, Turtushikh Damba, Yuehui Liang, Xue Liang, Jie Ma, Yunxiao Liang,** and **Yixing Li,** and **Lei Zhou:** Writing—review and editing. All authors have read the final manuscript and approved it for publication.

## CONFLICT OF INTEREST STATEMENT

The authors declare no conflicts of interest.

## ETHICS STATEMENT

All experimental protocols and procedures were approved by the Animal Experimentation Ethics Committee of Guangxi University (GXU‐2023‐0105). Patients or the public were not involved in the design, or conduct, or reporting, or dissemination plans of our research.

## Supporting information


**Figure S1.** HLF regulates intestinal lipid accumulation and absorption.
**Figure S2.** Partial *Hlf* deficiency improves energy metabolism.
**Figure S3.** *Hlf* knockout improves metabolic‐associated fatty liver disease (MAFLD).
**Figure S4.** *Hlf* Knockout Improves Energy Metabolism.
**Figure S5.** Intestinal Partial *Hlf* Deficiency Improves Lipid Digestion and Absorption in Mice.
**Figure S6.** Inhibition of PPARα Improves Energy Metabolism.
**Figure S7.** Inhibition of PPARα Attenuates Oxidative Stress.
**Figure S8.** The HLF and PPARα Axis Regulates Lipid metabolism through gut microbiota‐derived extracellular vesicles (fEVs).
**Figure S9.** Gut microbiota‐derived extracellular vesicles (fEVs) alleviate lipid metabolism dysregulation.
**Figure S10.** Gut microbiota‐derived extracellular vesicles (fEVs) from GW6471‐treated mice improve energy Metabolism.

**Table S1.** Summary of primer sequences.
**Table S2.** Summary of antibodies application.

**Movie S1.** HLF and PPARα axis regulates metabolic‐associated fatty liver disease through extracellular vesicles derived from the intestinal microbiota.

## Data Availability

The online public data used in this study (GSE188418 https://www.ncbi.nlm.nih.gov/geo/query/acc.cgi?acc=GSE188418). The sequencing data for the article has been uploaded to the GSA database (Project: PRJCA036195 https://bigd.big.ac.cn/gsa/browse/CRA023092). The data and scripts used are saved in GitHub https://github.com/yxz234/HL.git. Supplementary materials (methods, figures, tables, movies, graphical abstract, slides, videos, Chinese translated version, and update materials) may be found in the online DOI or iMeta Science http://www.imeta.science/.
