# Supplementary material for: HLF and PPARα axis regulates metabolic‐associated fatty liver disease through extracellular vesicles derived from the intestinal microbiota
Source: Imeta. 2025 Apr 7;4(2):e70022. doi: 10.1002/imt2.70022 (PMC11995174; doi:10.1002/imt2.70022)
Supplement: Supplementary file 1 — Figure S1. HLF regulates intestinal lipid accumulation and absorption. Figure S2. Partial Hlf deficiency improves energy metabolism. Figure S3. Hlf knockout improves metabolic‐associated fatty liver disease (MAFLD). Figure S4. Hlf Knockout Improves Energy Metabolism. Figure S5. Intestinal Partial Hlf Deficiency Improves Lipid Digestion and Absorption in Mice. Figure S6. Inhibition of PPARα Improves Energy Metabolism. Figure S7. Inhibition of PPARα Attenuates Oxidative Stress. Figure S8. The HLF and PPARα Axis Regulates Lipid metabolism through gut microbiota‐derived extracellular vesicles (fEVs). Figure S9. Gut microbiota‐derived extracellular vesicles (fEVs) alleviate lipid metabolism dysregulation. Figure S10. Gut microbiota‐derived extracellular vesicles (fEVs) from GW6471‐treated mice improve energy Metabolism. [file IMT2-4-e70022-s001.docx]

# Supporting information to HLF and PPARα axis regulates metabolic-associated fatty liver disease through extracellular vesicles derived from the intestinal microbiota

Running title**:** Hepatic leukemia factor regulates metabolic-associated fatty liver disease

Xingzhen Yang^1^, Jiale Wang^1^, Xinyu Qi^1^, Menglong Hou^2^, Mengkuan Liu^1^, Yang Xiao^2^, Siqi Liu^2^, Jinfeng Zhou^1^, Jingsu Yu^2^, Yang Wang^1^, Guo Chen^3^, Lin Yu^2^, Khongorzul Batchuluun^4,5^, Batbold Batsaikhan^5,6^, Turtushikh Damba^7^, Yuehui Liang^2^, Xue Liang^2^, Jie Ma^1^, Yunxiao Liang^2^, Yixing Li^1*^, Lei Zhou^2*^

^1^Guangxi Key Laboratory of Animal Breeding, Disease Control and Prevention, College of Animal Science and Technology, Guangxi University, Nanning, 530004, China.

^2^Institute of Digestive Disease, Guangxi Academy of Medical Sciences, the People’s Hospital of Guangxi Zhuang Autonomous Region, Nanning, 530021, China.

^3^Wincon TheraCells Biotechnologies Co., Ltd., Nanning, 530003, China.

^4^Center for Research and Development of Institute of Biomedical Sciences, Mongolian National University of Medical Sciences, Ulaanbaatar, 14120, Mongolia.

^5^Department of Health Research, Graduate School, Mongolian National University of Medical Sciences, Ulaanbaatar, 14120, Mongolia.

^6^Department of Internal Medicine, Institute of Medical Sciences, Mongolian National University of Medical Sciences, Ulaanbaatar, 14120, Mongolia.

^7^School of Pharmacy, Mongolian National University of Medical Sciences, Ulaanbaatar, 14120, Mongolia.

*Correspondence: [liyixing39@gxu.edu.cn](mailto:liyixing39@gxu.edu.cn) (Yixing Li), [lzhou@gxams.org.cn](mailto:%20lzhou@gxams.org.cn) (Lei Zhou).

## METHODS

### 1.1 Animal Experiments

Hepatic leukemia factor *(Hlf)* transgenic mice (*Hlf*^+/+^) and mice expressing Cre recombinase under the control of an intestinal Cre promoter were purchased from Cyagen Biosciences (Guangzhou, China). *Hlf*^+/+^ mice were crossed with intestinal Cre mice to generate intestinal-specific heterozygous *Hlf* knockout (*Hlf*^+/-^) mice and intestinal-specific homozygous *Hlf* knockout (*Hlf*^-/-^) mice, with *Hlf*^+/+^ mice serving as controls. At 8 weeks of age, the mice were divided into four groups (n = 6–9 per group) with no significant difference in mean body weight among groups: (1) *Hlf*^+/+^ fed a normal diet (*Hlf*^+/+^ ND), (2) *HLF*^+/-^ fed a normal diet (*Hlf*^+/-^ ND), (3) *Hlf*^+/+^ fed a high-fat diet (*Hlf*^+/+^ HFD), and (4) *Hlf*^+/-^ fed a high-fat diet (*Hlf*^+/-^ HFD). Body composition was recorded weekly for 14 weeks after diet intervention. At 22 weeks of age (following 14 weeks of diet intervention), mice were fasted for 12 hours and euthanized by cervical dislocation. At 8 weeks of age, the mice were divided into two groups (n = 5-6 per group) with no significant difference in mean body weight among groups: (1) *Hlf*^+/+^ fed a high-fat diet (*Hlf*^+/+^ HFD) and (2) *Hlf*^-/-^ fed a high-fat diet (*Hlf*^-/-^ HFD). At 22 weeks of age (following 14 weeks of diet intervention), mice were fasted for 12 hours and euthanized by cervical dislocation. Blood was collected from the retro-orbital sinus, and liver tissue, epididymal fat, and subcutaneous fat were harvested.

Male C57BL/6 mice (6 weeks old) were obtained from a commercial breeding facility (Beijing SPF Biotechnology Co., Ltd.). At 8 weeks of age, the mice were randomly divided into two groups (6–8 mice per group): (1) the HFD group, fed a high-fat diet (HFD, 5.5 kcal/g) and gavaged with saline; (2) the HFD+GW6471 group, fed HFD and gavaged with GW6471 (10 mg/kg body weight) (MedChemExpress, Monmouth Junction, NJ, USA). After 4 weeks of high-fat feeding, starting at 12 weeks of age, GW6471 was administered via oral gavage every other day for 8 weeks.

In a separate experiment, male C57BL/6 mice (6 weeks old) were purchased from the same supplier. At 8 weeks of age, they were randomly divided into two groups (n = 6–8 per group): (1) the HFD+CON fEVs group, and (2) the HFD+GW6471 fEVs group. Both groups were fed HFD, and fEVs (1 μg of total protein) were administered via oral gavage every other day for 12 weeks.

Throughout the experiments, the mice had ad libitum access to food and water and were maintained under standard laboratory conditions (temperature: 25 ± 2°C, humidity: 50 ± 5%, and a 12-hour light/dark cycle).

### 1.2 Genotyping

Genomic DNA was extracted from mouse tail samples and amplified by PCR using primers listed in Supplementary Table 1. The PCR products were separated by agarose gel electrophoresis, and the gel was visualized using a gel imaging system (Tocan, Shanghai, China).

### 1.3 Mouse Body Composition Analysis

Body composition was analyzed using a Niumag NMR analyzer (QMR23-060H-I, Suzhou, China). The instrument was calibrated using standard samples. After weighing the mice, they were placed into the detection tube, and their body weight was entered into the system. The body fat composition of the mice was then measured and analyzed.

### 1.4 Glucose Tolerance Test (GTT) and Insulin Tolerance Test (ITT)

For the glucose tolerance test (GTT), mice were fasted for 16 hours, followed by an intraperitoneal injection of glucose solution (1.5 g/kg). Blood glucose levels were measured at 0, 15, 30,60,90, and 120 minutes post-injection.

For the insulin tolerance test (ITT), mice were fasted for 4 hours, followed by an intraperitoneal injection of insulin solution (1 U/kg). Blood glucose levels were measured at 0, 15, 30,60,90, and 120 minutes post-injection.

### 1.5 Postprandial Triglyceride (TG) and Free Fatty Acid (FFA) Level Measurement

After a 16-hour fast, mice were orally gavaged with olive oil (10 μL/g body weight). Blood samples were collected from the tail vein at 0, 1, 2, 3, and 4 hours to measure serum triglyceride (TG) and free fatty acid (FFA) levels.

In the second week, mice were fasted for 16 hours and then administered an intraperitoneal injection of tyloxapol (500 mg/kg; MedChemExpress, Monmouth Junction, NJ, USA), followed by oral gavage with olive oil (10 μL/g body weight). Blood samples were collected from the tail vein at 0, 1, 2, 3, and 4 hours to measure serum TG and FFA levels.

### 1.6 Assessment of Intestinal Permeability in Mice

After a 16-hour fast, mice were orally gavaged with FITC-dextran (4 kDa, 1 g/kg; Sigma, Missouri, USA). Blood samples were collected 4 hours later, and fluorescence intensity was measured using a microplate reader. Intestinal permeability was quantified based on a standard curve of FITC-dextran.

### 1.7 Metabolic Cage Analysis

Metabolic cages (Sable Systems International, North Las Vegas, Nevada, USA) were used to monitor oxygen consumption (O₂), carbon dioxide production (CO₂), respiratory exchange ratio (RER), and energy expenditure. During the experiment, mice were maintained under a 12-hour light/12-hour dark cycle with ad libitum access to food and water.

### 1.8 Micro-Computed Tomography (Micro-CT)

Mice were anesthetized with tribromoethanol and then subjected to *in vivo* scanning using a micro-CT scanner (SkyScan 1278, Bruker, Billerica, Massachusetts, USA). Micro-CT scan results were obtained using reconstruction (NRecon) and analysis software (Dataviewer, CTVox, CTAN).

### 1.9 Histological Analysis

The liver and intestinal tissues of the sacrificed mice were placed in general tissue fixative (Servicebio, Wuhan, China) and stored at 4°C, while epididymal fat and subcutaneous fat were placed in a specialized fat fixative (Servicebio, Wuhan, China) and stored at 4°C. The samples were sent to Servicebio (Wuhan, China) for paraffin embedding, sectioning, and staining, and images were captured using an optical microscope. Additionally, mouse liver tissues were fixed in electron microscopy fixative (Servicebio, Wuhan, China) and stored at 4°C. The samples were then sent to Servicebio (Wuhan, China) for transmission electron microscopy analysis.

### 1.10 Fecal Extracellular Vesicle Isolation

Fresh feces were collected from 20-week-old CON mice, GW6471 mice, and *Hlf*^+/+^HFD and *Hlf*^+/^**^-^**HFD mice, and dissolved in PBS. The samples were then subjected to sequential centrifugation at 700 × g, 4000 × g, and 8,000 × g for 15 minutes each at 4°C. The supernatants were collected and filtered through a 0.45 μm filter. The filtrates were then ultracentrifuged at 100,000 × g for 120 minutes at 4°C, and the resulting pellets underwent a second ultracentrifugation. The supernatant was discarded, and the gut microbiota-derived extracellular vesicles (fEVs) were resuspended in PBS filtered through a 0.22 μm filter. Use of EVs serum-free medium (Umibio, Shanghai, Co., Ltd.) in subsequent cellular assays.

### 1.11 Transmission Electron Microscopy

First, 10 μL of gut microbiota-derived extracellular vesicles (fEVs) were placed on a copper grid and allowed to settle for 1 minute, after which excess liquid was absorbed with filter paper. Then, 10 μL of uranyl acetate was added to the copper grid and allowed to settle for 1 minute, with excess liquid again absorbed by filter paper. After air-drying for a few minutes, the sample was examined using a transmission electron microscope (HT-7700, Hitachi, Japan) at 100 kV for imaging.

### 1.12 Nanoparticle Tracking Analysis

Nanoparticle tracking analysis was performed using a NanoFCM (N30E, Xiamen, China). After instrument calibration using standard samples, an appropriate amount of fEVs was diluted in a gradient and loaded onto the device to determine the particle size and concentration of the fEVs.

### 1.13 *In Vivo* Tracking of fEVs

fEVs were incubated with Cy7.5 NHS ester (MedChemExpress, Monmouth Junction, NJ, USA) at room temperature for 6 hours, followed by concentration and purification using a 30 kDa ultrafiltration tube. Fluorescently labeled fEVs (20 μg total protein) were administered to each mouse via oral gavage. At 3, 6, 12, and 24 hours post-administration, *in vivo* imaging was performed using the IVIS-MARS system (Hangzhou, China). At 24 hours, the mice were euthanized, and imaging was conducted on the heart, liver, spleen, kidneys, and intestines.

### 1.14 Cell Culture

HepG2, Caco-2, and 293T cells (ATCC, Virginia, USA) were cultured in DMEM medium containing 10% fetal bovine serum (FBSSR-01021, OriCell, Cyagen Biosciences, Guangzhou, Inc) and 1% penicillin-streptomycin. For high-fat induction, cells were cultured in DMEM containing 0.4% oleic acid (OA), 0.2% palmitic acid (PA), 1% penicillin-streptomycin, and 2% bovine serum albumin. Cells were transfected with pcDNA3.1(-)-HLF(Hum) plasmid or siRNA (Table S1) according to the manufacturer's protocol, using a mixture of empty vector and liposomes (Yesen, Shanghai, China). The liposome complexes were transfected into Caco-2 cells cultured in serum-free DMEM. After 4-6 hours, the medium was replaced with OA/PA. After 48 hours, cell samples were collected.

### 1.15 Primary Hepatocyte Isolation

Male mice at 8 weeks of age were anesthetized intraperitoneally with 2.4% tribromoethanol and then euthanized. The inferior vena cava was cannulated with a 23 G needle, and the liver was perfused with 30 mL of perfusion buffer (1× HBSS without Ca²⁺, Mg²⁺, supplemented with 0.5 mM EDTA and 25 nM HEPES) (Solarbio, Beijing, China). The liver was then digested with 25 mL of digestion buffer (1× HBSS with Ca²⁺, Mg²⁺, 1 μg/mL Type II collagenase (Sigma-Aldrich,USA), and 1 nM HEPES) at 37°C. After perfusion, the liver was gently excised and filtered through a 70 μm nylon mesh and 10 mL of pre-chilled complete medium (DMEM supplemented with 10% FBS) at 4°C. Hepatocytes were isolated by centrifugation at 50 g for 2 minutes at 4°C. After discarding the supernatant, the hepatocytes were resuspended in 10 mL of complete medium containing 5 mL of freshly prepared 90% Percoll solution (9 mL Percoll and 1 mL PBS) (Solarbio, Beijing, China). To remove cell debris, the suspension was centrifuged at 200 g for 10 minutes at 4°C. The supernatant was aspirated, and the cells were resuspended and centrifuged again at 50 g for 2 minutes at 4°C. The cells were then resuspended in DMEM containing 1% glutamine and 1% penicillin-streptomycin and transferred to a culture plate for incubation at 37°C with 5% CO₂. The cells were maintained in the culture medium for 24 hours before being used in subsequent experiments.

### 1.16 Biochemical Assays for Tissues/Cells

Triglycerides (TG), total cholesterol (TC), aspartate aminotransferase (AST), alanine aminotransferase (ALT), low-density lipoprotein cholesterol (LDL-C), high-density lipoprotein cholesterol (HDL-C), and glutathione (GSH) assay kits were purchased from Nanjing Jianchen Bioengineering Institute (NJJCBIO, China). Free fatty acids (FFA) assay kit was purchased from Solarbio (Solarbio, Beijing, China), total iron (Fe) and ferrous ions (Fe²⁺) assay kits were purchased from Elabscience (Elabscience Biotechnology Co., Ltd). Malondialdehyde (MDA), catalase (CAT), superoxide dismutase (SOD), and ATP assay kits were purchased from Beyotime (Shanghai, China). The mouse reactive oxygen species (ROS) ELISA kit was purchased from Shanghai Enzyme-linked Biotechnology Co., Ltd. (Meilian, Shanghai, China), while the mouse lipopolysaccharide-binding protein (LBP) and cluster of differentiation 14 (sCD14) ELISA kits were purchased from Wuxi Donglin Technology Development Co., Ltd. (DLdevelop, Wuxi, China). The mouse lipopolysaccharide (LPS), lipid peroxides/lactoperoxidase (LPO), and taurochenodeoxycholic acid (TCDCA) ELISA kits were purchased from Jiangsu Enzyme-Linked Immunosorbent Assay. (Jiangsu, Meimian, Industrial, Co, Ltd). All procedures were strictly followed according to the manufacturer’s instructions.

### 1.17 Cell Fluorescence Staining and Quantification

All cell fluorescence staining and quantification procedures were strictly followed according to the instructions provided by the reagent manufacturers. Images were captured using an inverted fluorescence microscope (IX53; Olympus Corporation, Tokyo, Japan), and fluorescence intensity was measured using a microplate reader (Tecan, Männedorf, Switzerland).

MitoTracker Green FM mitochondrial green fluorescence probe (Yeasen, Shanghai, China), DCFH-DA green fluorescence probe (Beyotime, Shanghai, China), MitoSOX red fluorescence probe (Thermo Fisher, Waltham, MA), Calcein AM green fluorescence probe (Beyotime, Shanghai, China), FerroOrange red fluorescence probe (Dojindo), and 5-chloromethylfluorescein diacetate (CMFDA) fluorescence probe (Yeasen, Shanghai, China) were used for staining. The quantification of fluorescence staining for these six probes was standardized by cell protein concentration measured using a BCA protein assay kit (Beyotime, Shanghai, China).

Lipid peroxidation fluorescence staining was performed using the BODIPY 581/591 C11 fluorescence probe (MedChemExpress, Monmouth Junction, NJ, USA). Mitochondrial membrane potential (∆Ψm) fluorescence staining was performed using the mitochondrial membrane potential (JC-1) fluorescence probe (Beyotime, Shanghai, China). Quantification of these two fluorescence stains was expressed as the red fluorescence intensity/green fluorescence intensity, as detected by the microplate reader.

### 1.18 Fatty Acid Uptake Assay

Caco-2 cells were seeded in clear-bottom black 96-well flat plates, and the assay was conducted according to the manufacturer's instructions (MAK156, Sigma-Aldrich, USA).

### 1.19 FITC-Dextran Permeability Assay in Caco-2 Monolayers

Caco-2 cells were seeded in 12-well transwell plates (LABSELECT, Hefei, China) and differentiated for 21 days. FITC-dextran 4 and FITC-dextran 40 (4 kDa and 40 kDa; Sigma-Aldrich, USA) solutions (1 mg/mL) were added to the apical chamber of the monolayer. Basolateral media were collected, and FITC levels were measured using a fluorescence reader and standard curve.

### 1.20 Dual-Luciferase Reporter Assay

The transcription start site of peroxisome proliferator-activated receptor alpha *(Ppara)* was identified using the eukaryotic promoter database, with a region of 2000 base pairs upstream of the transcription start site. The retrieved sequence was verified through NCBI. The upstream sequence was then analyzed using the JASPAR core database to identify potential HLF binding sites. Mutations were introduced at the predicted binding sites, and a pGL3-Basic vector was used to construct the firefly luciferase reporter plasmid. 293-T cells were seeded in 24-well culture plates and cultured overnight. When cell density reached 70%, the cells were transfected and co-transfected. After 48 hours post-transfection, dual-luciferase reporter gene assays were performed using the Dualucif® Firefly & Renilla Detection Kit (US EVERBRIGHT, Suzhou, China). Renilla luciferase was used to normalize transfection efficiency and luciferase activity.

### 1.21 Gut microbiota-derived extracellular vesicles (fEVs) Uptake Experiment

Following the manufacturer's protocol, fEVs were labeled with Dil (Yeasen, Shanghai, China), and PBS was used as a control sample. Cells were cultured in media without EVs until they reached 60% confluence. The purified Dil-labeled fEVs were incubated with cells at a concentration of 10 µg/ml for 12 hours. After incubation, cells were washed three times with PBS and fixed with 2% paraformaldehyde. Visualization was performed using an inverted fluorescence microscope, and cell nuclei were stained with DAPI (Sercicebio, Wuhan, China).

### 1.22 Western Blot

The fEVs, cells, or tissue samples were lysed using RIPA buffer (LABLEAD, lnc, Beijing, China) at 4°C, followed by centrifugation at 12,000 rpm for 10 minutes. The protein concentration was measured using the BCA Protein Assay Kit (Beyotime, Shanghai, China). The supernatant was mixed with 4× loading buffer (Solarbio, Beijing, China) at a 3:1 ratio and heated at 100°C for 10 minutes to prepare protein samples. Proteins were separated on an 8-15% pre-cast SDS-PAGE gel (Bio-Rad) and transferred to a nitrocellulose membrane according to the manufacturer’s instructions. The membrane was blocked with TBST containing 0.1% Tween-20 (Solarbio, Beijing, China) and 5% skim milk (Biosharp, Hefei, China) at room temperature for 1 hour. Primary antibodies against GPA33 (Bioss, Beijing, China), CD9 (Bioss, Beijing, China), CD81 (Bioss, Beijing, China), TSG101 (Bioss, Beijing, China), HLF (Proteintech, Wuhan, China), PPARα (ABclonal, Wuhan, China), ZO-1 (Bioss, Beijing, China), Occludin (Proteintech, Wuhan, China), ACSL4 (Huabio, Hangzhou, China), GPX4 (Huabio, Hangzhou, China), SLC7A11 (Huabio, Hangzhou, China), and α-Tubulin (Huabio, Hangzhou, China) were used, followed by HRP-conjugated secondary antibodies (anti-rabbit IgG or anti-mouse IgG, Jackson, PA, USA) to detect the bound proteins(Table S2).

### 1.23 Quantitative real-time PCR

According to the manufacturer's instructions, total RNA was extracted from cells or tissues using the standard TRIzol protocol, and then reverse transcribed into cDNA using the GeneAmp PCR Reagent Kit (Promega, Madison, WI, USA). Quantitative real-time PCR (qRT-PCR) was performed using specific gene primers (Table S1) and the Power SYBR Green PCR Master Mix (Genstar, Beijing, China).

### 1.24 Single-Cell Transcriptome Sequencing

After sampling the intestinal tissues of healthy and spontaneously fatty liver cynomolgus monkeys, the samples were sent to LC-Bio (Hangzhou, China) for single-cell transcriptome and bulk transcriptome sequencing and analysis, following the manufacturer's instructions. Bioinformatic analysis was performed using the OmicStudio tools at <https://www.omicstudio.cn/tool>.

### 1.25 Gut Microbiota 16S Sequencing

Fresh fecal samples were collected from 22-week-old CON and GW6471 mice fed a high-fat diet (HFD). The 16S rDNA gene was amplified using primers: 5′- CCTACGGGNGGCWGCAG -3′ and 5′- GGACTACHVGGGTATCTAAT -3′. Sequencing and statistical analysis were performed by GENEDENOVO (Guangzhou, China). Bioinformatic analysis was performed using the omicshare tools at <https://www.omicshare.com/tools/>.

### 1.26 Transcriptome Sequencing

Total RNA was extracted from mouse intestinal tissues using the standard TRIzol protocol. The extracted RNA was used for subsequent library construction and high-throughput sequencing. The cDNA library was constructed by Qingke (Beijing Tsingke Biotech CO., Ltd) and sequenced using the Illumina HiSeq 2000 platform. Bioinformatic analysis was performed using the omicshare tools at <https://www.omicshare.com/tools/>.

### 1.27 Lipidomics Sequencing

Fresh fEVs were extracted and sent in equal amounts to MetWare (Wuhan, China) for sequencing and statistical analysis, following the manufacturer's instructions. Bioinformatic analysis was performed using the Metware Cloud at <https://cloud.metware.cn>.

### 1.28 Statistical Analysis

All data are expressed as mean ± standard deviation (SD). Two-way repeated-measures analysis of variance (ANOVA) was used to compare trends across two curves over multiple time points. The Friedman test was used for four-group comparisons with repeated measures over time. Statistical comparisons between two groups were conducted using the Student’s t-test, while multiple group comparisons were performed using one-way ANOVA with GraphPad Prism 9.0 or SPSS 26.0. *P* value < 0.05 (*) or < 0.01 (**) was considered statistically significant. Different letters in the figure indicate significant differences between groups.

## Supplementary figures

**
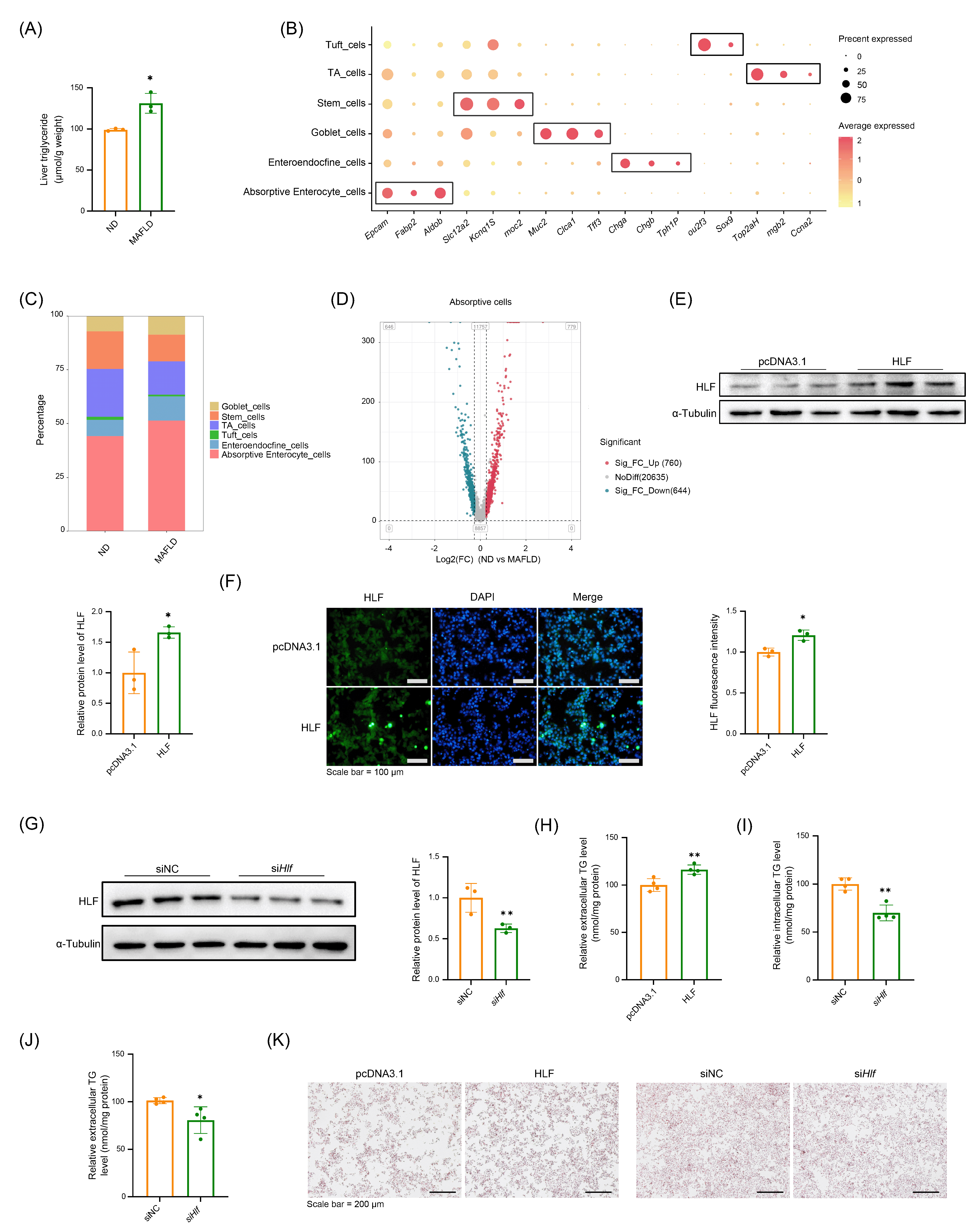
**

Figure S1. **Hepatic Leukemia Factor (HLF) Regulates Intestinal Lipid Accumulation and Absorption.** (A) Hepatic triglyceride (TG) levels in cynomolgus monkeys (*n* = 3). (B) Bubble plot of marker genes for cell types. (C) Bar graph of cell proportions. (D) Volcano plot of genes expressed in intestinal absorptive epithelial cells (metabolic-associated fatty liver disease (MAFLD) vs normal diet (ND)). (E) Immunoblot and quantification of HLF protein in Caco-2 cells overexpressing HLF (*n* = 3). (F)Immunofluorescence analysis of HLF overexpression in Caco-2 cells (*n* = 3). (G) Immunoblot and quantification of HLF protein in Caco-2 cells with HLF silencing (*n* = 3). (H–J) Intracellular and extracellular TG levels in Caco-2 cells with HLF overexpression or silencing (*n* = 4). (K) Oil Red O staining of Caco-2 cells with HLF overexpression or silencing. Data are presented as mean ± standard deviation. **p* < 0.05, ***p* < 0.01.


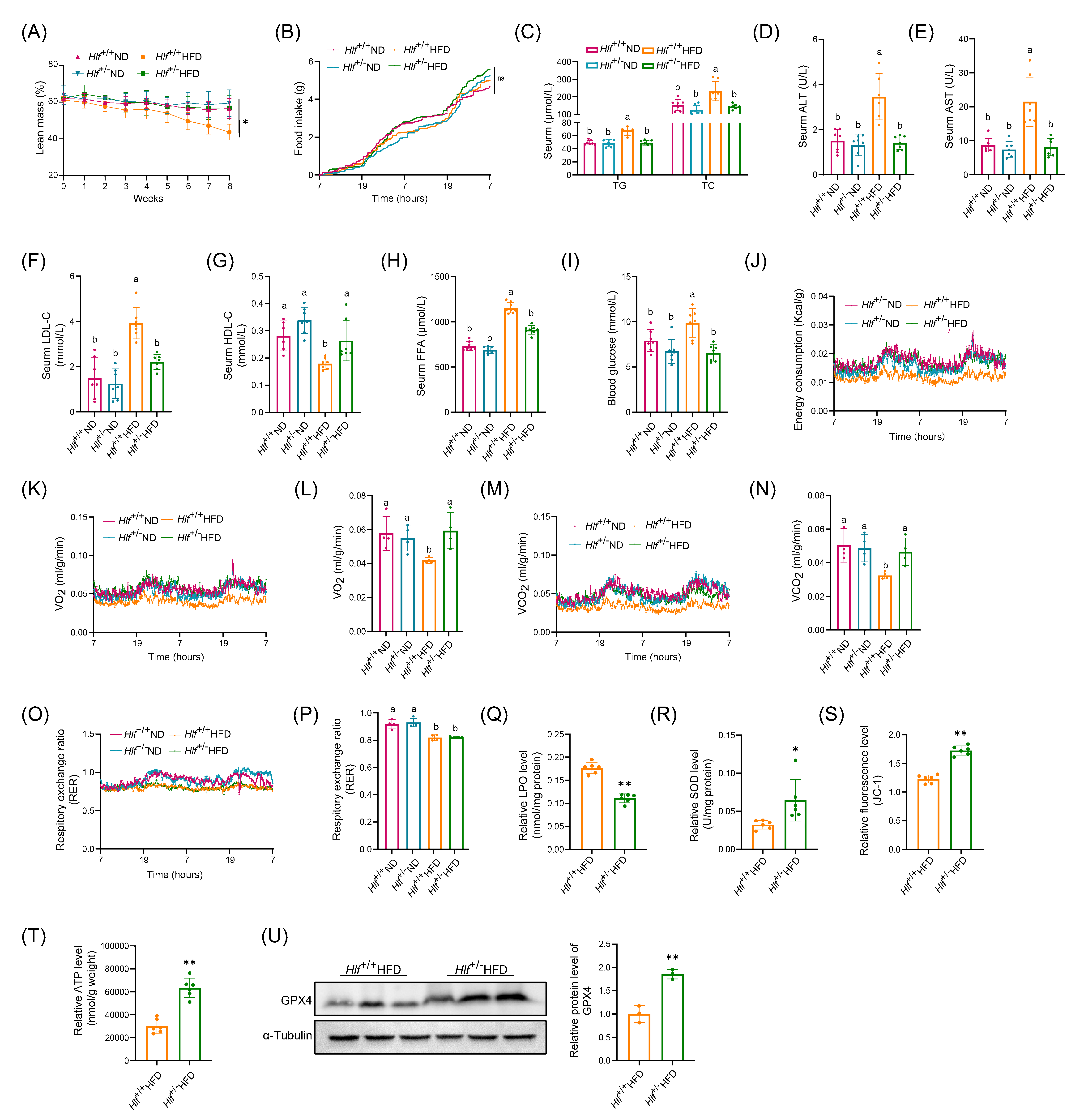


Figure S2. **Partial Hepatic Leukemia Factor (HLF) Deficiency Improves Energy Metabolism**. (A) Lean mass percentage in mice (*n* = 6). (B) Food intake in mice. (C) Serum triglyceride (TG) and total cholesterol (TC) levels in mice (*n* = 6). (D–H) Serum levels of alanine aminotransferase (ALT), aspartate aminotransferase (AST), low-density lipoprotein cholesterol (LDL-C), high-density lipoprotein cholesterol (HDL-C), and free fatty acids (FFA) in mice (*n* = 6). (I) Fasting blood glucose levels in mice (*n* = 6). (J–P) Oxygen consumption (O₂), carbon dioxide production (CO₂), and respiratory exchange ratio (RER, VO₂/VCO₂) in mice (*n* = 4). (Q–T) Liver levels of lipid peroxides/lactoperoxidase (LPO), superoxide dismutase (SOD), mitochondrial membrane potential (JC-1), and adenosine triphosphate (ATP) (*n* = 6). (U) Western blot analysis and quantification of **glutathione peroxidase 4 (**GPX4) in liver tissues (*n* = 3). Data are presented as mean ± standard deviation. The Friedman test was used for four-group comparisons with repeated measures over time. **p* < 0.05, ***p* < 0.01. Different letters in the figure indicate significant differences between groups.


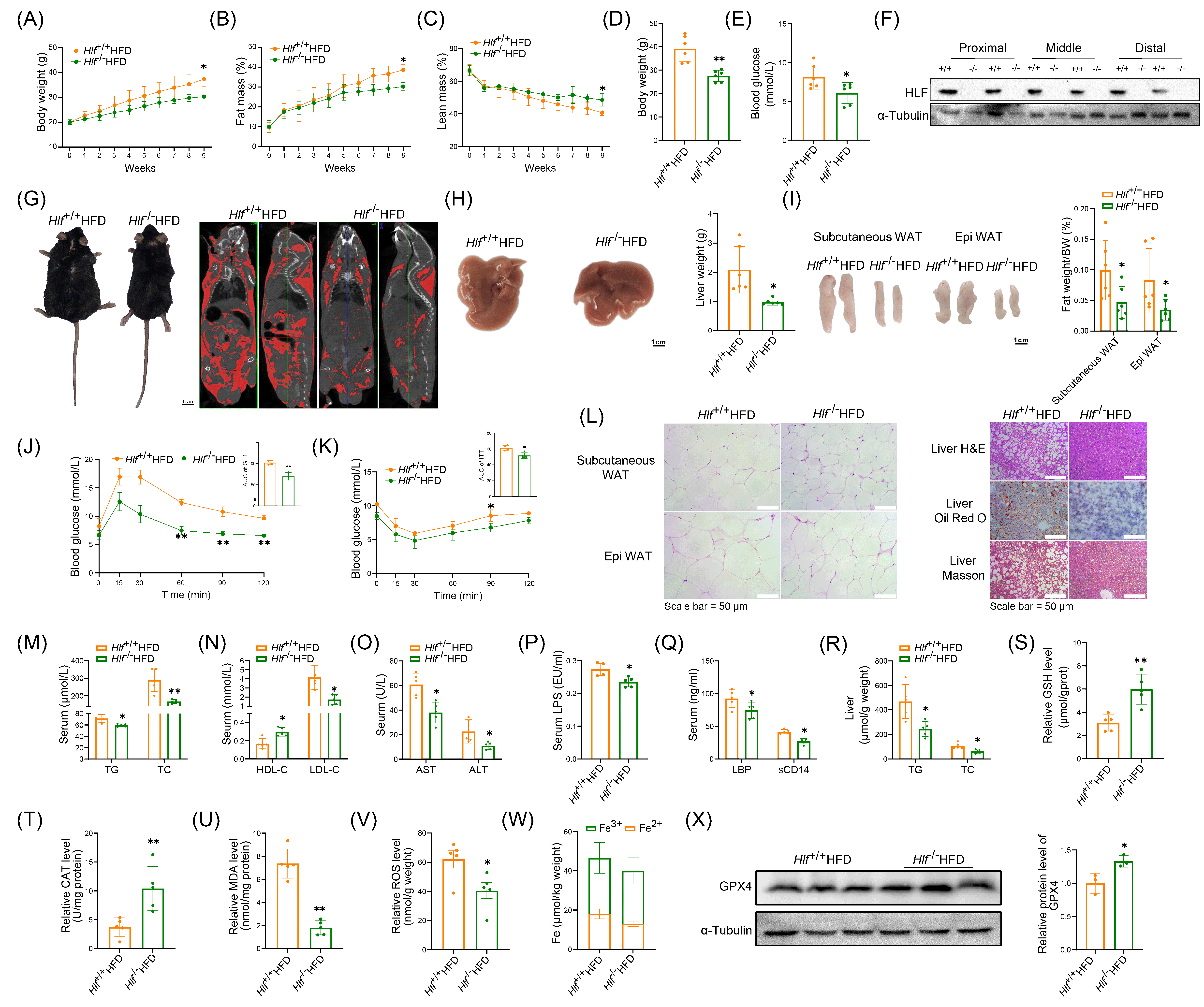


Figure S3. **Hepatic Leukemia Factor (HLF) Knockout Improves Metabolic-Associated Fatty Liver Disease (MAFLD)**. (A–C) Body weight, body fat percentage, and lean mass percentage in high-fat diet-fed mice (*n* = 6). (D) Body weight curve of mice. (E) Fasting blood glucose levels in mice (*n* = 5–6). (F) Western blot analysis of HLF expression in intestinal tissues (*n* = 3). (G) Representative mouse body size and computed tomography (CT) images. (H) Liver weight and representative liver images of mice. (I) Subcutaneous and epididymal fat weights along with representative images. (J) Glucose tolerance test (GTT) and quantification of AUC in mice (*n* = 6). (K) Insulin sensitivity test and quantification of AUC in mice (*n* = 6). (L) H&E staining of epididymal and subcutaneous fat, and H&E, Oil Red O, and Masson staining of the liver. (M) Serum triglyceride (TG) and total cholesterol (TC) levels (*n* = 5). (N) Serum high-density lipoprotein cholesterol (HDL-C) and low-density lipoprotein cholesterol (LDL-C) levels (*n* = 5). (O) Serum aspartate aminotransferase (AST) and alanine aminotransferase (ALT) levels (*n* = 5). (P) Serum lipopolysaccharide (LPS) levels (*n* = 5). (Q) Serum lipopolysaccharide-binding protein (LBP) and cluster of differentiation 14 (sCD14) levels (*n* = 5). (R) Hepatic TG and TC levels (*n* = 5). (S–W) Hepatic levels of glutathione (GSH), catalase (CAT), Malondialdehyde (MDA), reactive oxygen species (ROS), and Fe²⁺/Fe³⁺ (*n* = 5). (X) Western blot analysis and quantification of **glutathione peroxidase 4** (GPX4) in liver tissues (*n* = 3). Data are presented as mean ± standard deviation. Repeated measures analysis of variance was used to compare trends across two curves over multiple time points. **p* < 0.05, ***p* < 0.01.


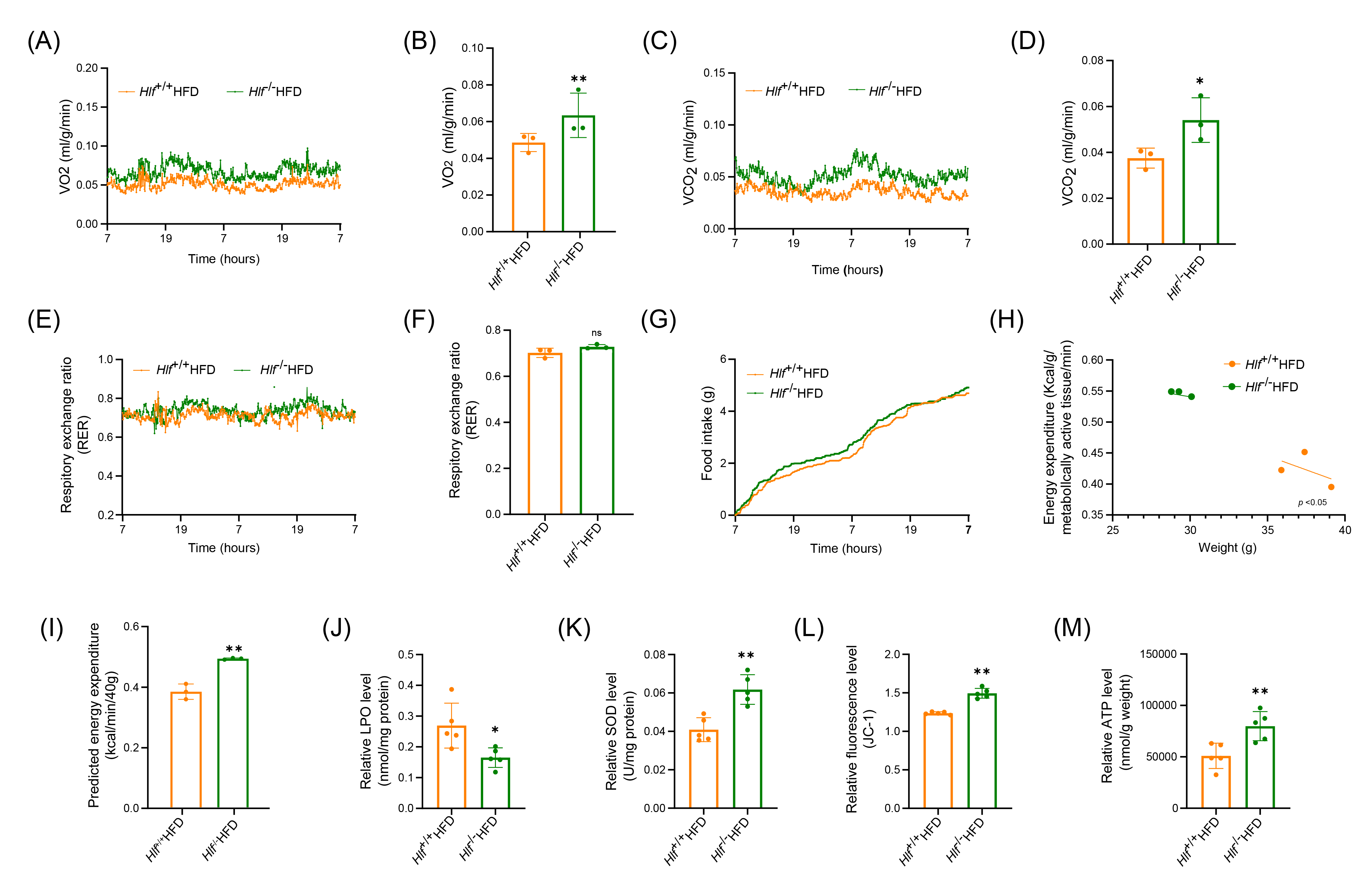


Figure S4. **Hepatic Leukemia Factor (HLF) Knockout Improves Energy Metabolism.** (A–B) Oxygen consumption (O₂). (C–D) Carbon dioxide production (CO₂). (E–F) Respiratory exchange ratio (RER, VO₂/VCO₂). (G) Food intake. (H–I) Energy metabolism analysis in mice (*n* = 3). (J–M) Liver levels of lipid peroxides/lactoperoxidase (LPO), superoxide dismutase (SOD), mitochondrial membrane potential (JC-1), and adenosine triphosphate (ATP) (*n* = 5). Data are presented as mean ± standard deviation. Repeated measures analysis of variance was used to compare trends across two curves over multiple time points. **p* < 0.05, ***p* < 0.01.


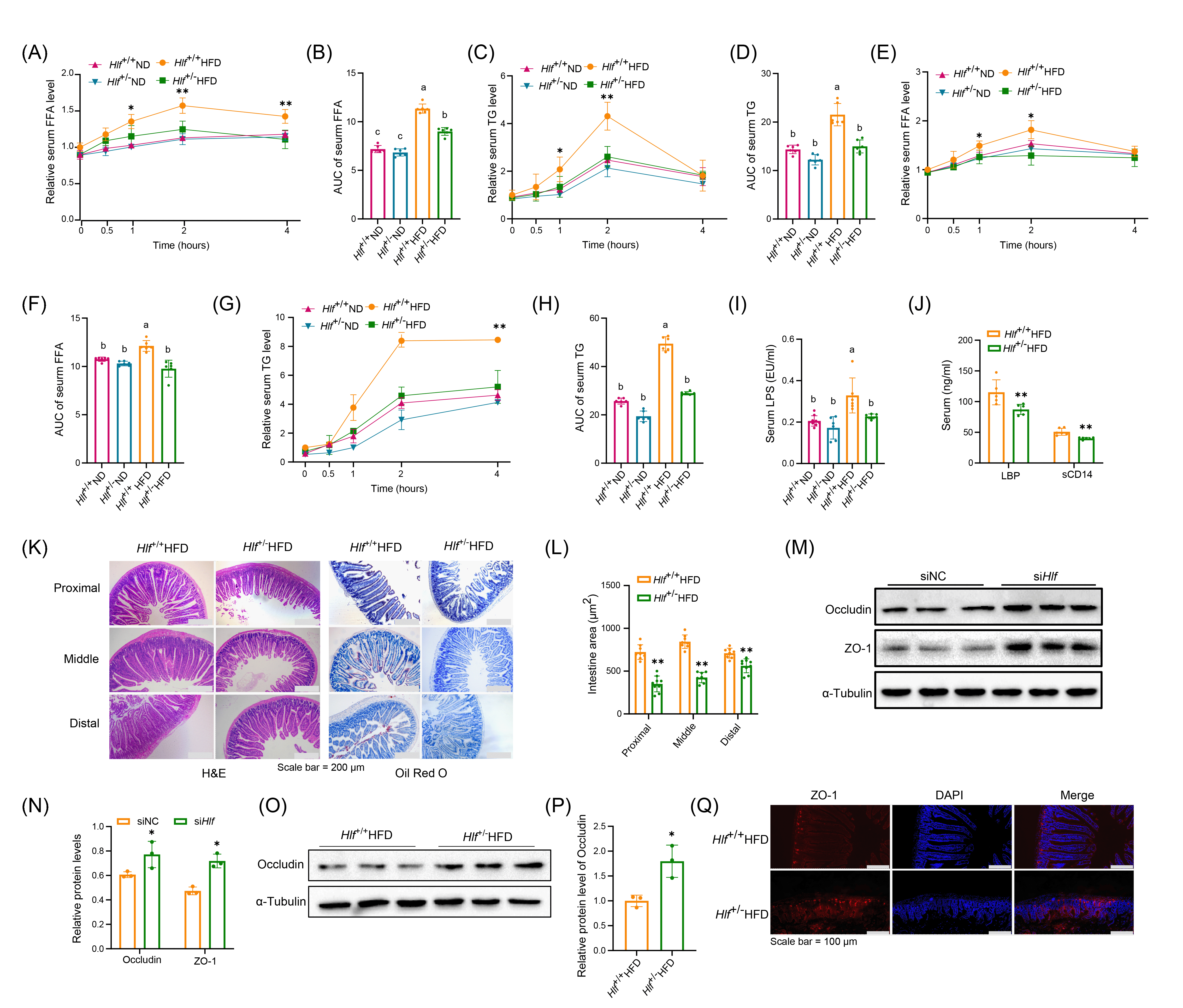


Figure S5. **Intestinal Partial Hepatic Leukemia Factor (HLF) Deficiency Improves Lipid Digestion and Absorption in Mice.** (A–B) Serum free fatty acids (FFA) levels and area under the curve (AUC) from 0 to 4 hours in mice (*n* = 6). (C–D) Serum triglyceride (TG) levels and AUC from 0 to 4 hours in mice (*n* = 6). (E–F) Serum FFA levels and AUC from 0 to 4 hours after Tyloxapol injection in mice (*n* = 6). (G–H) Serum TG levels and AUC from 0 to 4 hours after Tyloxapol injection in mice (*n* = 6). (I–J) Serum levels of lipopolysaccharide (LPS), lipopolysaccharide-binding protein (LBP)*,* and cluster of differentiation 14 (sCD14) in mice (*n* = 6). (K–L) H&E and Oil Red O staining of different intestinal sections and quantification of villus area (*n* = 8–10). (M–N) Western blot analysis and quantification of zonula occludens-1 (ZO-1) and Occludin expression after HLF silencing (*n* = 3). (O–P) Western blot analysis and quantification of Occludin expression in the intestinal tissues of mice (*n* = 3). (Q) Immunofluorescence staining of ZO-1 in the intestinal tissues of mice. Data are presented as mean ± standard deviation. The Friedman test was used for four-group comparisons with repeated measures over time. **p* < 0.05, ***p* < 0.01. Different letters in the figure indicate significant differences between groups.


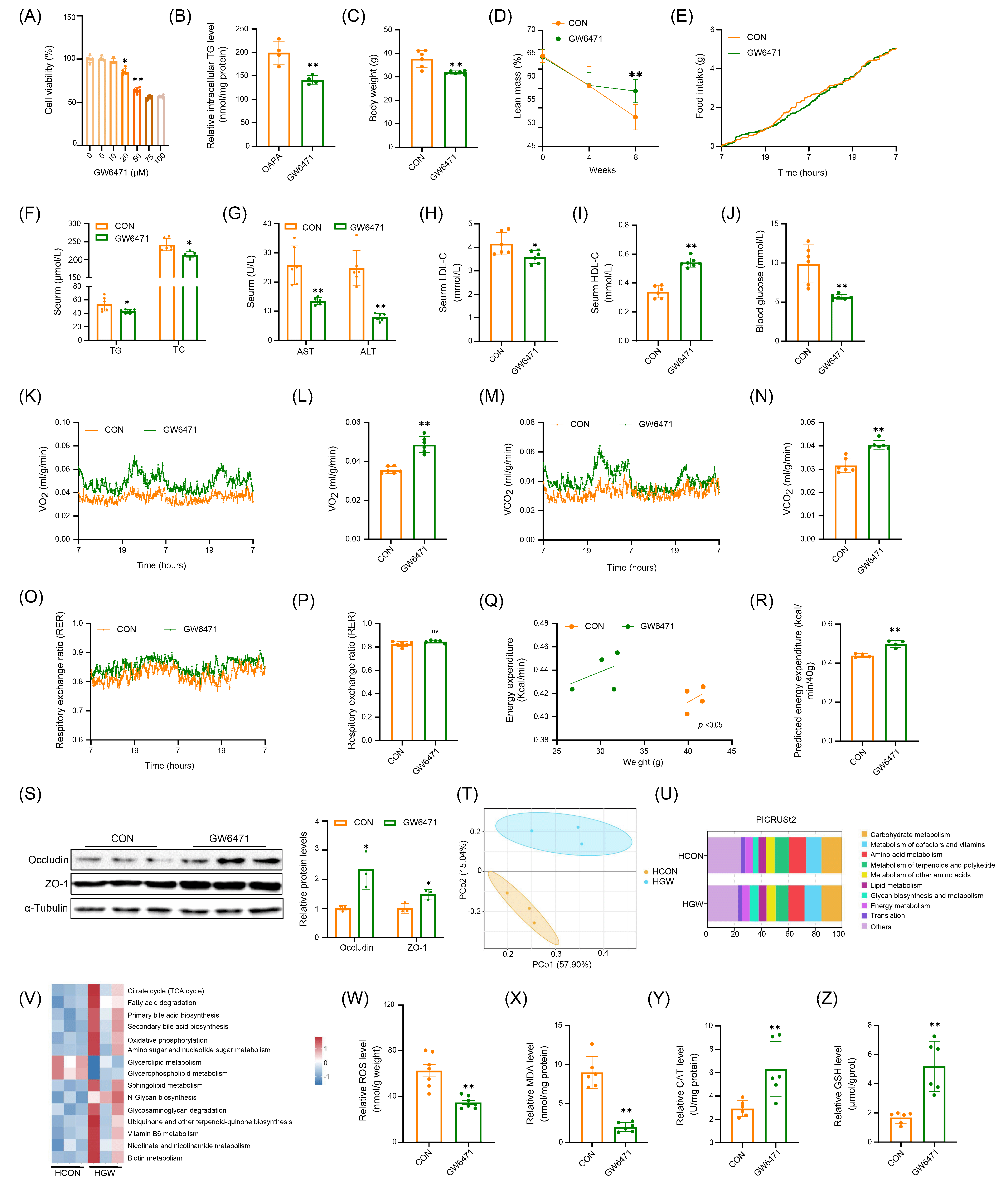


Figure S6. **Inhibition of Peroxisome Proliferator-Activated Receptor Alpha** (**PPARα) Improves Energy Metabolism.** (A) Cell viability (CCK8) assay in Caco-2 cells (*n* = 4). (B) Intracellular triglyceride (TG) levels in Caco-2 cells (*n* = 4). (C–D) Body weight and lean mass ratio of mice (*n* = 6). (E) Food intake of mice. (F) Serum TG and total cholesterol (TC) levels in mice (*n* = 6). (G–I) Serum levels of aspartate aminotransferase (AST), alanine aminotransferase (ALT), low-density lipoprotein cholesterol (LDL-C), and high-density lipoprotein cholesterol (HDL-C) in mice (*n* = 6). (J) Fasting blood glucose levels in mice (*n* = 6). (L–R) Oxygen consumption (O₂), carbon dioxide production (CO₂), respiratory exchange ratio (RER, VO₂/ VCO₂), energy metabolism analysis, and normalized energy expenditure (to 40 g body weight) in mice (*n* = 4). (S) Western blot analysis and quantification of zonula occludens-1 (ZO-1) and Occludin in intestinal tissues (*n* = 3). (T) PCoA plot of gut microbiota based on 16S rRNA sequencing. (U–V) Functional prediction stack diagram and pathway heatmap of gut microbiota. (W–Z) Liver levels of reactive oxygen species (ROS), malondialdehyde (MDA), catalase (CAT), and glutathione (GSH) (*n* = 6). Data are presented as mean ± standard deviation. Repeated measures analysis of variance was used to compare trends across two curves over multiple time points. **p* < 0.05, ***p* < 0.01.


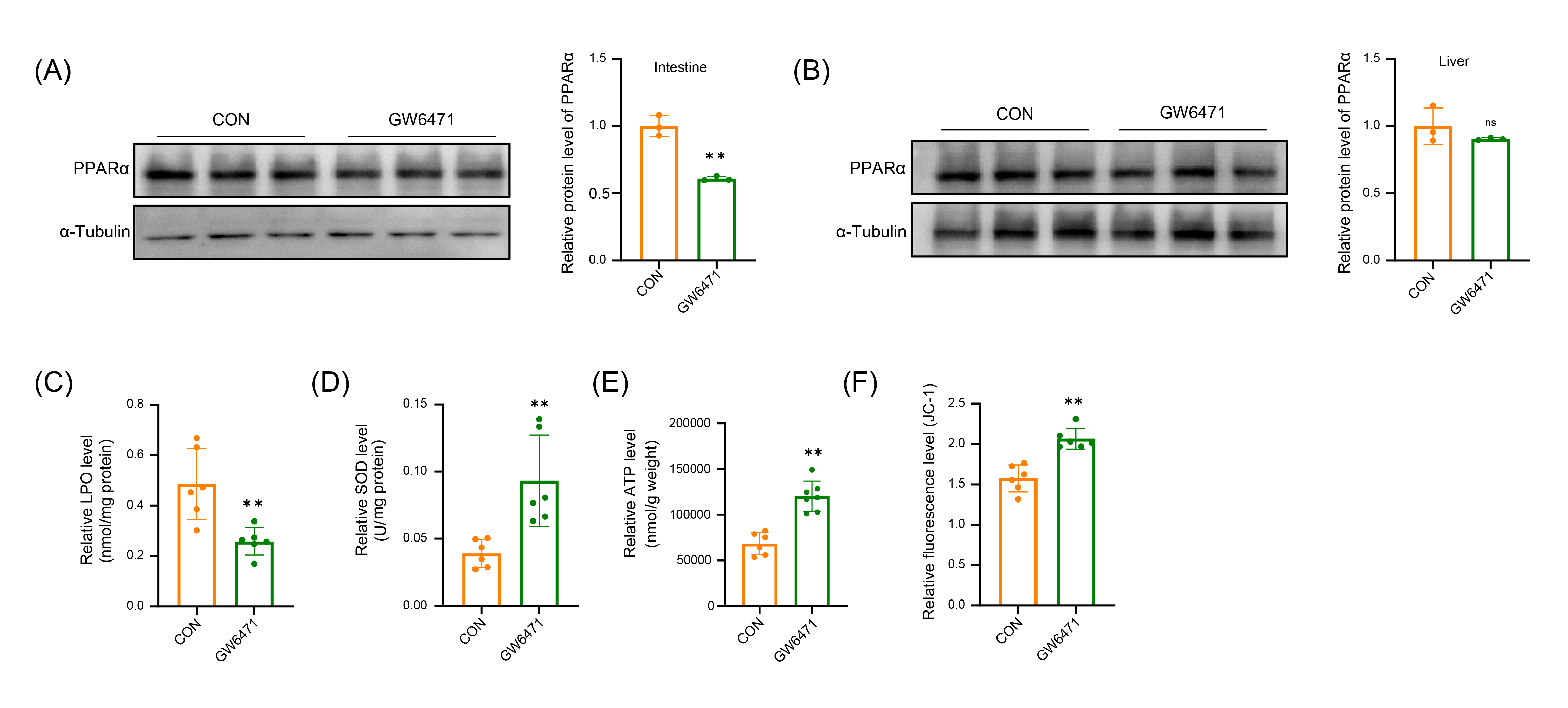


Figure S7. **Inhibition of Peroxisome Proliferator-Activated Receptor Alpha** (**PPARα)** **Attenuates** **Oxidative Stress.** (A–B) Western blot analysis and quantification of **PPARα** in intestinal and liver tissues (*n* = 3). (C–F) Hepatic levels of Liver levels of lipid peroxides/lactoperoxidase (LPO), superoxide dismutase (SOD), mitochondrial membrane potential (JC-1), and adenosine triphosphate (ATP) (*n* = 6). Data are presented as mean ± standard deviation. **p* < 0.05, ***p* < 0.01.


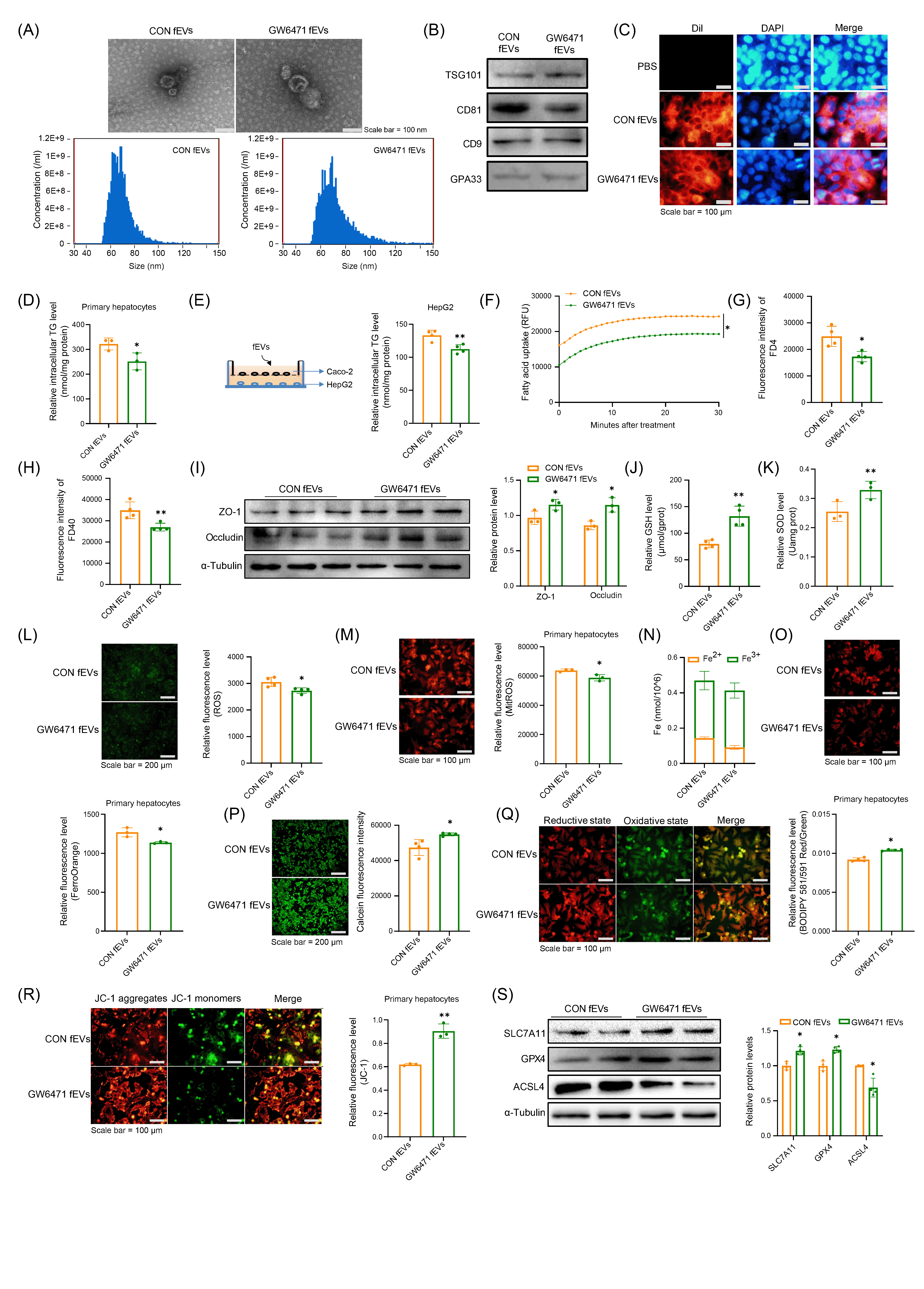


Figure S8. **The Hepatic Leukemia Factor (HLF) and** **Peroxisome Proliferator-Activated Receptor Alpha** (**PPARα) Axis Regulates Lipid Metabolism Through Gut Microbiota-Derived Extracellular Vesicles (fEVs).** (A) Transmission electron microscopy (TEM) and nanoparticle tracking analysis (NTA) of fecal extracellular vesicles (fEVs). (B) Western blot analysis of extracellular vesicle marker proteins. (C) Cellular uptake of intestinal fEVs. (D) Intracellular triglyceride (TG) levels in primary hepatocytes (*n* = 3). (E) Schematic diagram of cell co-culture and intracellular TG levels in the lower chamber cells (*n* = 4). (F) Fatty acid uptake in Caco-2 cells (*n* = 4). (G–H) 4 kDa fluorescein isothiocyanate-dextran (FD4) and 40 kDa fluorescein isothiocyanate-dextran (FD40) permeability levels in Caco-2 cells (*n* = 4). (I) Western blot analysis and quantification of ZO-1 and Occludin in Caco-2 cells (*n* = 3). (J–L) Levels of glutathione (GSH), superoxide dismutase (SOD), and reactive oxygen species (ROS) in HepG2 cells (*n* = 3–4). (M) Mitochondrial ROS (MitROS) levels in primary hepatocytes (*n* = 3). (N) Fe²⁺ levels in HepG2 cells (*n* = 4). (O) FerroOrange fluorescence levels in primary hepatocytes (*n* = 3). (P) Calcein green fluorescence levels in HepG2 cells (*n* = 4). (Q–R) Lipid peroxidation and mitochondrial membrane potential (JC-1) levels in primary hepatocytes (*n* = 3–4). (S) Western blot analysis and quantification of solute carrier family 7 member 11 (SLC7A11), **glutathione peroxidase 4** (GPX4), and acyl-coa synthetase long chain family member 4 (ACSL4) in HepG2 cells (*n* = 3). Data are presented as mean ± standard deviation. Repeated measures analysis of variance was used to compare trends across two curves over multiple time points. **p* < 0.05, ***p* < 0.01.


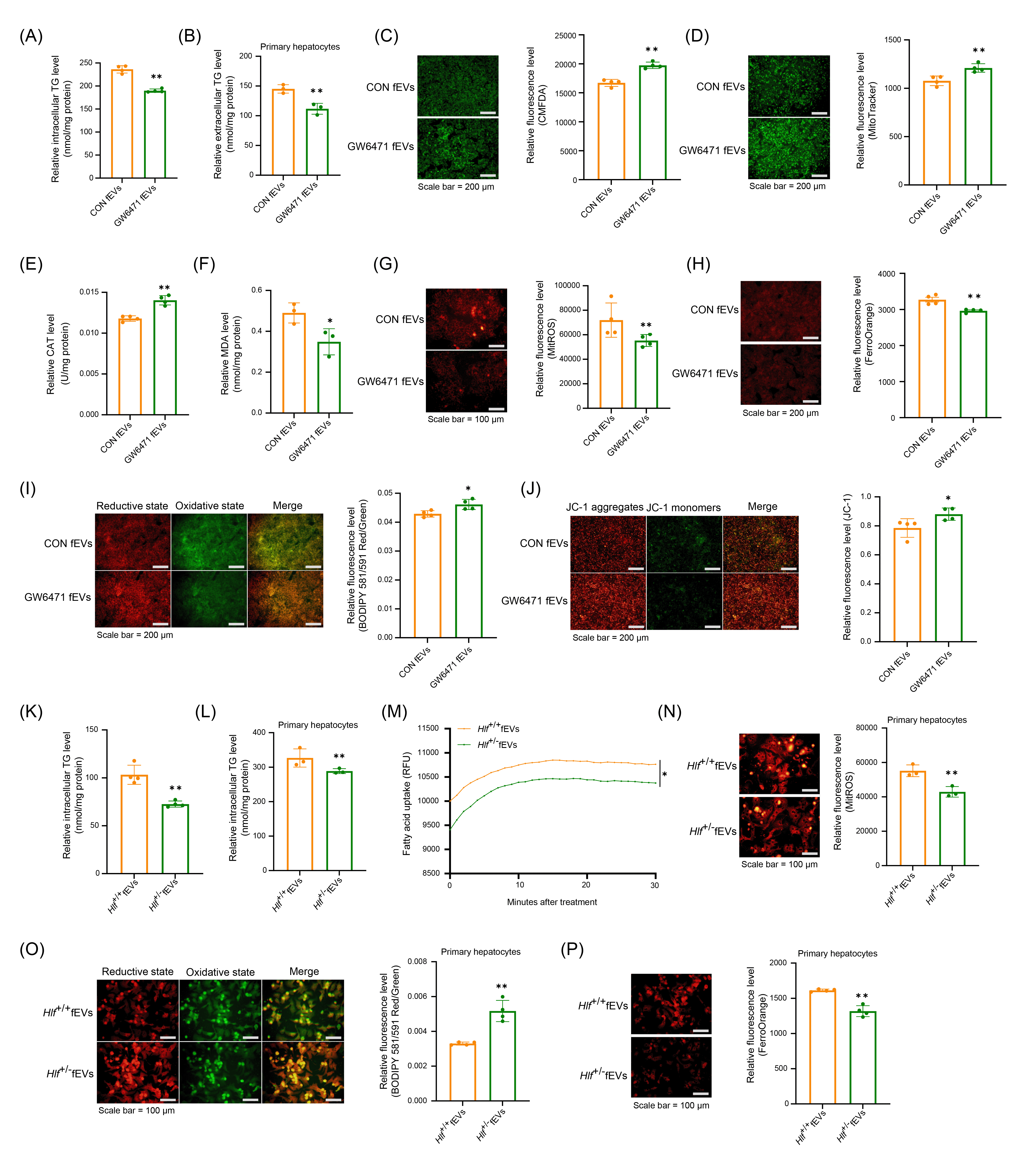


Figure S9. **Gut Microbiota-Derived Extracellular Vesicles (fEVs) Alleviate Lipid Metabolism Dysregulation.** (A) Intracellular triglyceride (TG) levels in HepG2 cells (*n* = 4). (B) Extracellular TG levels in primary hepatocytes (*n* = 3). (C) CMFDA fluorescence detection in HepG2 cells in the lower chamber under co-culture conditions (*n* = 4). (D) Mitochondrial staining in HepG2 cells (*n* = 4). (E–F) Levels of catalase (CAT) and malondialdehyde (MDA) in HepG2 cells (*n* = 3–4). (G–J) Mitochondrial reactive oxygen species (MitROS), FerroOrange fluorescence, lipid peroxidation, and mitochondrial membrane potential (JC-1) in HepG2 cells (*n* = 4). (K–L) Intracellular TG levels in HepG2 cells and primary hepatocytes (*n* = 3–4). (M) Fatty acid uptake in Caco-2 cells (*n* = 4). (N–P) Mitochondrial ROS, lipid peroxidation, and FerroOrange fluorescence levels in primary hepatocytes (*n* = 4). Data are presented as mean ± standard deviation. Repeated measures analysis of variance was used to compare trends across two curves over multiple time points. **p* < 0.05, ***p* < 0.01.


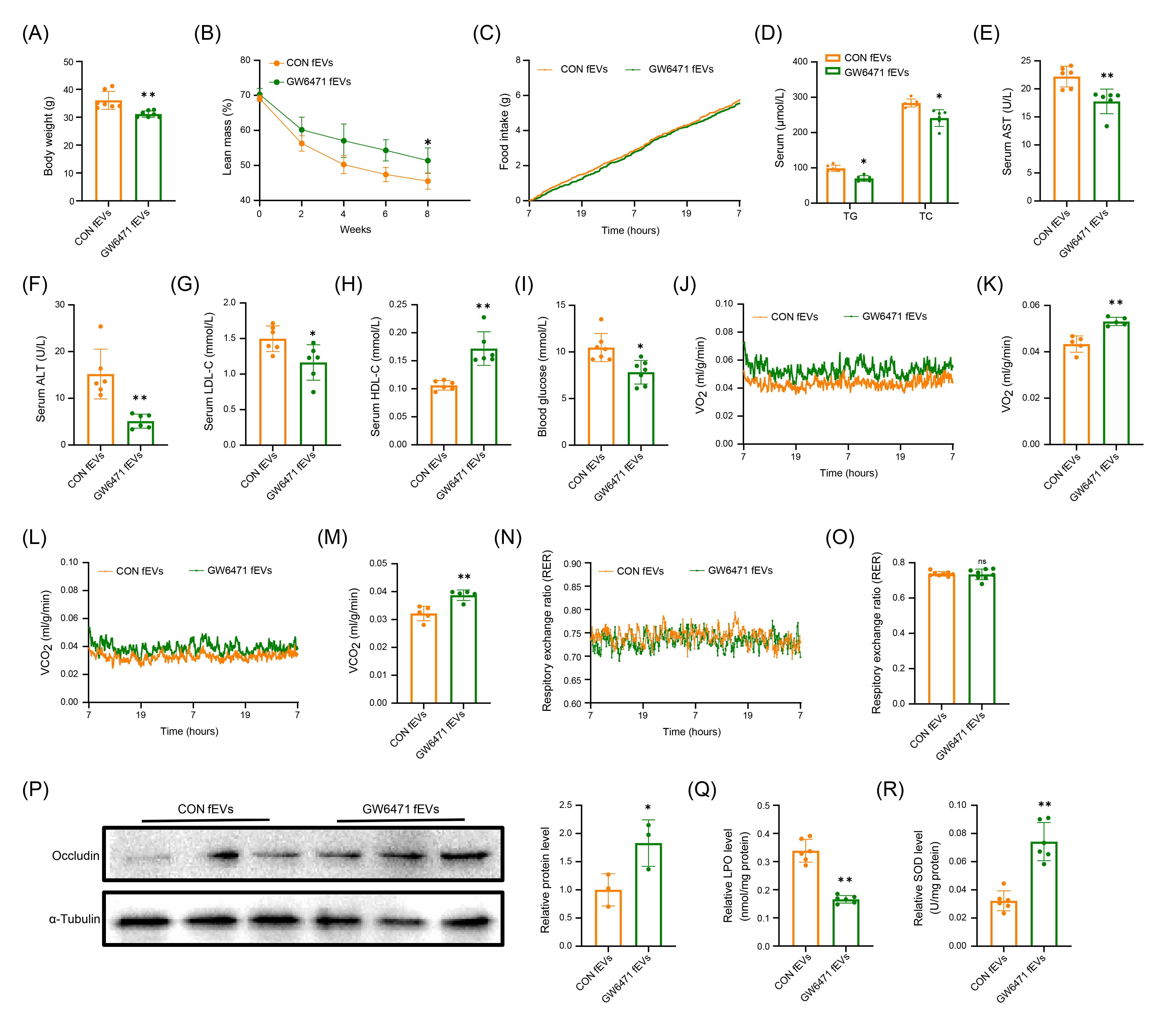


Figure S10. **Gut Microbiota-Derived Extracellular Vesicles (fEVs) from GW6471-treated mice Improve Energy Metabolism.** (A–B) Body weight and lean mass percentage of mice (*n* = 6). (C) Food intake of mice. (D) Serum triglyceride (TG) and total cholesterol (TC) levels in mice (*n* = 6). (E–H) Serum aspartate aminotransferase (AST), alanine aminotransferase (ALT), low-density lipoprotein cholesterol (LDL-C), and high-density lipoprotein cholesterol (HDL-C) levels in mice (*n* = 6). (I) Fasting blood glucose levels in mice (*n* = 6). (J–O) Oxygen consumption (O₂), carbon dioxide production (CO₂), and respiratory exchange ratio (RER, VO₂/CO₂) in mice (*n* = 5). (P) Western blot analysis and quantification of Occludin in intestinal tissues (*n* = 3). (Q–R) Hepatic levels of lipid peroxides/lactoperoxidase (LPO) and superoxide dismutase (SOD) (*n* = 6). Data are presented as mean ± standard deviation. Repeated measures analysis of variance was used to compare trends across two curves over multiple time points. **p* < 0.05, ***p* < 0.01.
